# Recommended nursing care for people with mental disorders in mobile prehospital care: a scoping review

**DOI:** 10.1590/0034-7167-2024-0595

**Published:** 2025-12-08

**Authors:** Liliane Pena, Richardson Miranda Machado, Deborah Franscielle da Fonseca, Maria Candida Brandão Valadares Bicalho, Larissa Martins Santos, Patrícia Peres de Oliveira

**Affiliations:** I Universidade Federal de São João del-Rei. Divinópolis, Minas Gerais, Brazil

**Keywords:** Nursing Care, Mental Disorders, Mental Health Assistance, Patient Safety, Emergency Medical Services., Atención de Enfermería, Trastornos Mentales, Atención a la Salud Mental, Seguridad del Paciente, Servicios Médicos de Urgencia.

## Abstract

**Objectives:**

to map scientific evidence regarding recommended nursing care for individuals with mental disorders in mobile prehospital care.

**Methods:**

a scoping review, according to the JBI methodology. Research conducted in seven databases, eight catalogs of theses and dissertations, national guidelines/protocols, and international guidelines, in any language and without time limit.

**Results:**

of the 4,184 publications, 23 were selected and comprised the final sample. Four domains were identified (safety and protection of people with mental disorders, professionals and third parties; assessment of individuals with mental disorders; management of individuals with mental disorders; important aspects regarding mechanical restraint), subdivided into 27 nursing care practices.

**Conclusions:**

the nursing care recommended for people with mental disorders in mobile pre-hospital care included risk prevention, effective communication, scene management, behavioral management, attention to suicidal emergencies, as well as aspects related to mechanical restraint and non-pharmacological and pharmacological interventions.

## RESUMEN

**Objetivos:**

mapear la evidencia científica sobre la atención de enfermería recomendada para personas con trastornos mentales en atención prehospitalaria móvil.

**Métodos:**

revisión exploratoria, según la metodología del JBI. Investigación realizada en siete bases de datos, ocho catálogos de tesis y disertaciones, guías/protocolos nacionales y guías internacionales, en cualquier idioma y sin límite de tiempo.

**Resultados:**

de las 4.184 publicaciones, se seleccionaron 23, que constituyeron la muestra final. Se identificaron cuatro dominios (seguridad y protección de la persona con enfermedad mental, profesionales y terceros; evaluación de la persona con enfermedad mental; manejo de la persona con sufrimiento mental; aspectos importantes de la contención mecánica), subdivididos en 27 prácticas de atención de enfermería.

**Conclusiones:**

los cuidados de enfermería recomendados para personas con trastornos mentales en atención prehospitalaria móvil incluyeron prevención de riesgos, comunicación efectiva, manejo de la escena, manejo conductual, atención a emergencias suicidas, así como aspectos relacionados con la contención mecánica e intervenciones no farmacológicas y farmacológicas.

## INTRODUCTION

Mental disorders affect approximately 18.0% of the global population, causing a substantial health-related burden worldwide^(1)^. Although the recognition of mental health disorders demands healthcare services, significant treatment gaps remain. To date, substantial barriers such as a lack of professionals and resources, negative perceptions, and stigma hinder help-seeking behaviors^(1,2)^.

All countries have seen the effects of the COVID-19 pandemic on health and social and emotional well-being. As a result of this emotional impact on global mental health, there has been a significant increase in the number of people experiencing psychological distress worldwide^(1,3-5)^. As a consequence, there has been an increase in emergency calls for mobile pre-hospital care (MPHC) involving people with mental disorders, which has required emergency nursing professionals to carry out psychiatric crisis assessments, with the appropriate knowledge and skills^(6-11)^.

From this perspective, mental healthcare in Brazil has been undergoing important changes. Currently, it is dedicated to the qualification, implementation, and strengthening of the Psychosocial Care Network established by Ordinance 3,088/2011^(12)^, republished on May 21, 2013, and revoked by Consolidation Ordinance of September 28, 2017^(13)^, which deals with the consolidation of the standards on Healthcare Networks within the Brazilian Health System as a manifestation of the political process of implementing the Psychiatric Reform and the Brazilian National Mental Health Policy^(14)^.

Thus, mental healthcare is guided by the perspective of Healthcare Networks, which direct it through clinical and organizational guidelines. Emergency care centers are responsible, within their scope of operation, for the reception, risk assessment, and care in emergency situations of people with mental disorders and needs arising from the use of crack, alcohol, and other drugs. These include Basic Health Units, stabilization rooms, hospital emergency care/emergency rooms, the Emergency Care Unit, and MPHC^(12,13)^.

Within the Emergency Care Network^(15)^, MPHC in Brazil, carried out by the Mobile Emergency Care Service (In Portuguese, *Serviço de Atendimento Móvel de Urgência* - SAMU), aims to coordinate the flow of care and provide early assistance and appropriate, rapid, and effective transportation to victims, including those with mental disorders. However, studies indicate that, in relation to psychiatric emergency care, the service habitually reproduces the execution of attitudes that go against the Psychiatric Reform and the Brazilian National Mental Health Policy, such as physical and chemical restraints carried out incorrectly and/or on occasions when they were not essential for care^(2,16)^.

On the other hand, assistance to people in mental health emergencies and urgent situations needs to be safe, based on scientific evidence, using algorithms that include assessment, diagnosis and management, in addition to being seen as an opportunity to provide support and promote dialogue^(2,14)^.

Despite the advancement of knowledge about patient safety, literature still lacks evidence and mapping of recommended nursing care for people with mental distress in MPHC settings^(17)^. It should be noted that a preliminary search was conducted in PROSPERO, the Medical Literature Analysis and Retrieval System Online (MEDLINE) via the National Library of Medicine and National Institutes of Health (PubMed), the Cochrane Database of Systematic Reviews, and the JBI Evidence Synthesis, and no completed or ongoing reviews with this focus were identified. Therefore, this scoping review may contribute to nursing practice in primary care, in addition to potentially impacting health policies and the safety of care for people with mental disorders.

Given the above, it is important to generate evidence that can support original studies and synthesize information regarding psychic emergencies in MPHC, in order to provide support for safe and qualified nursing practice.

Undoubtedly, the permanent improvement of the quality of pre-hospital care needs to be developed based on solid evidence recognized in the scientific field, in order to provide a systematic and coordinated approach by managers and interventionist nursing professionals.

## OBJECTIVES

To map scientific evidence regarding the nursing care recommended for people with mental disorders in MPHC.

## METHODS

### Ethical aspects

The use of public domain data and the non-involvement of human beings excluded the need for assessment by the Research Ethics Committee.

### Study design

This study is a scoping review, which consists of mapping the main concepts related to a research area as well as clarifying working definitions and/or conceptual limitations of a topic based on available evidence, aiming to obtain comprehensive results^(18-20)^.

To ensure rigor, this scoping review was guided by the JBI review methodology^(20)^, considering the Preferred Reporting Items for Systematic reviews and Meta-Analyses for Scoping Reviews checklist recommendations^(19)^, in addition to registering its protocol on the Open Science Framework platform, with DOI identification:10.17605/OSF.IO/M58GT.

To conduct the study, nine stages were followed: 1) objective definition and alignment and research question identification; 2) inclusion and exclusion criteria development with the objective; 3) description of planned approach for evidence search, selection, data extraction, and presentation of evidence; 4) evidence investigation; 5) evidence selection; 6) evidence extraction; 7) evidence analysis; 8) presentation of results; and 9) summary of evidence in relation to the review objective, with observation of the implications discovered^(20)^.

### Guiding question and search

To construct the research question, the Participants, Concept, and Context (PCC)^(20)^ strategy was used, in which P (Participants) - people with mental disorders, C (Concept) - recommended nursing care, and C (Context) - SAMU. The following research question emerged: what nursing care is considered recommended for people with mental disorders in the context of MPHC?

Based on the research question, a search was initially conducted in MEDLINE via PubMed and the Cumulative Index to Nursing and Allied Health Literature (CINAHL) database to identify the main descriptors of the Medical Subject Headings and Health Sciences Descriptors as well as keywords used in studies addressing the topic of interest. It should be noted that the search strategy was developed with the input of a health sciences research librarian.

After selecting descriptors and synonyms, an electronic search for studies was conducted in the MEDLINE via PubMed, Latin American and Caribbean Literature in Health Sciences (LILACS), CINAHL, Scopus, Embase, Web of Science, and the Cochrane Library electronic databases. Gray literature included the Catalog of Theses and Dissertations of *Coordenação de Aperfeiçoamento de Pessoal de Nível Superior* (CAPES), National Library of Australia (Trove), Academic Archive Online (DiVA), The DART-Europe E-Theses Portal, the National ETD Portal for South African Theses and Dissertations, *Red de Repositorios Latinoamericano*, World Cat Dissertations and Theses, and *Repositórios Científicos de Acesso Aberto de Portugal* (RCAAP), as well as national and international guidelines/protocols, with no time limit. Chart 1 shows the search strategy for studies/documents was structured.

**Chart 1:** Search strategies applied to each database and gray literature, Divinópolis, Minas Gerais, Brazil, 2024

Databases	Strategies
MEDLINE (via PubMed)	(Mental Health OR Mental Disorders OR Psychiatry OR Intellectual Disability OR Mental Health Services OR Mental Health Assistance OR Psychiatric Nursing OR Mental Disorders Nursing OR Mental Status and Dementia Tests OR Mentally Ill Persons OR United States Substance Abuse and Mental Health Services Administration OR Persons with Mental Disabilities OR Psychic Symptoms OR Cognitive Dysfunction OR Neurocognitive Disorders OR Substance-Related Disorders OR Crisis Intervention) AND (Patient Safety OR Medication Errors OR Safety culture) AND (Ambulances OR Emergency Mobile Units OR Mobile Emergency Units OR Mobile Emergency Service OR Emergency medical assistance service OR Mobile Health Units OR Prehospital Care OR Emergency Medical Services OR Emergency Medicine OR SAMU)
LILACS	(Mental Health) OR (Mental Disorders) AND (Patient Safety) OR (Medication Errors) OR (Safety culture) AND (Ambulances) OR (Emergency Mobile Units) OR (Mobile Emergency Units) OR (Mobile Emergency Service) OR (Emergency medical assistance service) OR (Prehospital Care) OR (Emergency Medical Services) OR (Mobile Health Units) OR (Emergency Medicine)
Scopus (via portal CAPES)	(TITLE-ABS-KEY (mental AND health) OR TITLE-ABS-KEY (mental AND disorders) OR TITLE-ABS-KEY (psychiatric AND nursing) OR TITLE-ABS-KEY (mental AND disorders AND nursing) AND TITLE-ABS-KEY (patient AND safety) OR TITLE-ABS-KEY (medication AND errors) OR TITLE-ABS-KEY (safety AND culture) AND TITLE-ABS-KEY (ambulances) OR TITLE-ABS-KEY (emergency AND mobile AND units) OR TITLE-ABS-KEY (prehospital AND care) OR TITLE-ABS-KEY (mobile AND emergency AND units)) AND PUBYEAR > 1991 AND PUBYEAR < 2024
Cochrane Library	Cochrane Reviews matching (Mental Health OR Mental Disorders OR Psychiatry OR Intellectual Disability OR Mental Health Services OR Mental Health Assistance OR Psychiatric Nursing OR Mental Disorders Nursing OR Mental Status and Dementia Tests OR Mentally Ill Persons OR United States Substance Abuse and Mental Health Services Administration OR Persons with Mental Disabilities OR Psychic Symptoms OR Cognitive Dysfunction OR Neurocognitive Disorders OR Substance-Related Disorders OR Crisis Intervention) AND (Patient Safety OR Medication Errors OR Safety culture) AND (Ambulances OR Emergency Mobile Units OR Mobile Emergency Units OR Mobile Emergency Service OR Emergency medical assistance service OR Mobile Health Units OR Prehospital Care OR Emergency Medical Services OR Emergency Medicine)
Web of Science	(Mental Health OR Mental Disorders OR Psychiatry OR Intellectual Disability OR Mental Health Services OR Mental Health Assistance OR Psychiatric Nursing OR Mental Disorders Nursing OR Mental Status and Dementia Tests OR Mentally Ill Persons OR United States Substance Abuse and Mental Health Services Administration OR Persons with Mental Disabilities OR Psychic Symptoms OR Cognitive Dysfunction OR Neurocognitive Disorders OR Substance-Related Disorders OR Crisis Intervention) AND (Patient Safety OR Medication Errors OR Safety culture) AND (Ambulances OR Emergency Mobile Units OR Mobile Emergency Units OR Mobile Emergency Service OR Emergency medical assistance service OR Mobile Health Units OR Prehospital Care OR Emergency Medical Services OR Emergency Medicine OR SAMU)
CINAHL	(“Mental Health” OR “Mental Disorders”) AND (“Patient Safety” OR “Safety culture”) AND (“Ambulances” OR “Emergency Mobile Units” OR “Mobile Emergency Units” OR “Prehospital Care” OR “Emergency Medical Services”)
Embase	(‘mental disease’:ti,ab,kw AND ‘patient safety’:ti,ab,kw OR ‘safety culture’:ti,ab,kw) AND ‘emergency care’:ti,ab,kw OR ambulances:ti,ab,kw
**Gray literature**	**Strategies**
The DART-Europe E-Theses Portal	(Mental Health OR Mental Disorders OR Psychiatry OR Psychiatric Nursing OR Substance-Related Disorders OR Crisis Intervention) AND (Patient Safety OR Medication Errors OR Safety culture) AND (Ambulances OR Emergency Mobile Units OR Mobile Emergency Units OR Mobile Emergency Service OR Emergency medical assistance service OR Mobile Health Units OR Prehospital Care)
CAPES	Ambulances AND Patient Safety/ Ambulances AND Mental Health/ Ambulances AND Mental Health AND Patient Safety
RCAAP	*Saúde Mental e Segurança do Paciente e Ambulâncias* *Saúde Mental e Segurança do paciente e Atendimento Pré-hospitalar* *Saúde Mental e Segurança do paciente e Assistência Pré-Hospitalar*
National ETD portal (EThOS)	(Mental Disorders OR Psychiatry) AND (Patient Safety OR Medication Errors OR Safety culture) AND (Ambulances OR Mobile Emergency Units OR Prehospital Care)
*Red de Repositorios Latinoamericanos*	(Mental Disorders OR Psychiatry) AND (Patient Safety OR Medication Errors OR Safety culture) AND (Ambulances OR Prehospital Care)
WorldCat dissertations and theses	(Mental Disorders OR Psychiatry) AND (Patient Safety OR Medication Errors OR Safety culture) AND (Ambulances OR Mobile Emergency Units OR Prehospital Care)
TROVE	(Mental Disorders OR Psychiatry) AND (Patient Safety OR Medication Errors OR Safety culture) AND (Ambulances OR Mobile Emergency Units OR Prehospital Care)
DiVA	(Mental Disorders OR Psychiatry) AND (Patient Safety OR Medication Errors OR Safety culture) AND (Ambulances OR Mobile Emergency Units OR Prehospital Care)
QAS	(Mental Disorders OR Psychiatry) AND (Patient Safety OR Medication Errors OR Safety culture) AND (Ambulances OR Prehospital Care)
NICE	(Mental Disorders OR Psychiatry) AND (Patient Safety OR Medication Errors OR Safety culture) AND (Ambulances OR Prehospital Care)
NHS	(Mental Disorders OR Psychiatry) AND (Patient Safety OR Medication Errors OR Safety culture) AND (Ambulances OR Prehospital Care)
NSW Ministry of Health (AU)	(Mental Disorders OR Psychiatry) AND (Patient Safety OR Medication Errors OR Safety culture) AND (Ambulances OR Prehospital Care)
Ministry of Health (BR)	*Saúde Mental e Segurança do paciente e Atendimento Pré-hospitalar* *Saúde Mental e Segurança do paciente e Assistência Pré-Hospitalar*

*NHS - National Health System; NICE - National Institute for Health and Care Excellence; TROVE - National Library of Australia; DiVA - Academic Archive Online; QAS - Queensland Ambulance Service; RCAAP - Repositórios Científicos de Acesso Aberto de Portugal; CAPES - Coordenação de Aperfeiçoamento de Pessoal de Nível Superior; CINAHL - Cumulative Index to Nursing and Allied Health Literature; LILACS - Literatura Latino-Americana e do Caribe em Ciências da Saúde; MEDLINE - Medical Literature Analysis and Retrieval System Online; PubMed - National Library of Medicine and National Institutes of Health.*

The searches were conducted on January 2, 2024, and a new search was established in all databases and sources in July 2024, through remote access to the databases, based on the CAPES journal portal registered via the Federated Academic Community. It is noteworthy that new studies were included due to the reverse analysis of study/document references.

### Inclusion and exclusion criteria

The study population consisted of research related to safe nursing care for people with mental disorders, carried out by nursing professionals at MPHC and published in full in any language and without a time limit.

Articles that did not address the research question, publications that did not present the abstract in the databases used, full texts unavailable online and articles that include other healthcare professionals working in MPHC, as well as abstracts, conference proceedings, comments, preliminary editorial notes, reviews, letters, experience reports, theoretical essays, single case studies and narrative reviews were excluded.

### Data selection, analysis and treatment

Study selection was conducted by two previously trained independent reviewers, and disagreements were resolved by a third reviewer. The selection process occurred in two stages. In the first, the titles and abstracts of references obtained through the search strategy were analyzed, preselecting potentially eligible studies. In the second stage, the full text of preselected studies was assessed to confirm their eligibility, i.e., a) confirming their relevance to the review question and, if applicable, b) extracting relevant data for later development of this study.

It should be noted that Mendeley Desktop version 1.19.2 was used as the bibliography manager. The results obtained were exported to the Rayyan^®^ reference manager, developed by the Qatar Computing Research Institute. The manager enabled the removal of duplicate documents, the selection and screening of documents by three researchers independently, and disagreements were resolved by consensus.

Information from the documents selected for analysis was extracted independently using Microsoft Excel^®^ spreadsheets. A pilot test of the structured data extraction form was also conducted for each type of evidence source^(20)^. A fourth reviewer participated in validating the information and in the discussion to establish consensus among the authors, when necessary.

After the pilot test, a group discussion with the scoping review authors was held in order to reach an agreement on all aspects of the tool, data to be extracted, and resolution of doubts and conflicts.

The structured form allowed for data synthesis, interpretation, and basic numerical analysis of the scope, nature, and distribution of studies. Items such as authors, year of publication, country of origin, objectives, place of publication, recommended nursing care for individuals with mental disorders in primary care, level of evidence, and full-text access link were grouped together. Thus, for each publication, the main aspects related to the study’s problem, context, methods, results, discussions, and conclusions were identified and extracted.

The level of evidence was assessed according to the JBI proposal^(21)^, considering: level 1: experimental studies (systematic review of randomized controlled trials, randomized controlled clinical trial, and pseudo-randomized controlled clinical trial); level 2: quasi-experimental studies (systematic review of quasi-experimental studies, systematic review of quasi-experimental studies and other lower-evidence designs, prospective quasi-experimental controlled study, pre-test, post-test, or control group study); level 3: observational analytical studies (systematic review of comparable cohort studies, systematic review of comparable cohorts and other lower-evidence study designs, cohort study with control group, case-control study, and observational studies without a control group); level 4: observational descriptive studies (systematic review of descriptive studies, cross-sectional study, case series, and case study); level 5: Expert opinion and bench research (systematic review of expert opinion, expert consensus, and bench research/expert opinion).

Information mapping was established based on the JBI instrument to characterize the productions^(20)^, and the studies/documents found were thoroughly analyzed. The results were synthesized through a thematic approach and presented descriptively through tables and charts.

The data were subsequently categorized into domains according to the nursing care recommended for individuals with mental disorders in MPHC. Based on the categorized data, a narrative presentation of the information was created.

## RESULTS

**Figure 1:**
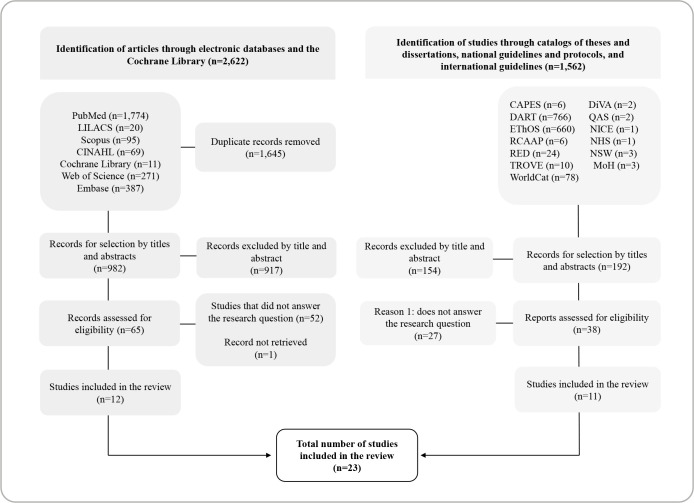
Flowchart of the study selection process for the scoping review adapted from the Preferred Reporting Items for Systematic reviews and Meta-Analyses for Scoping Reviews^(20)^, Divinópolis, Minas Gerais, Brazil, 2024

The data search generated a total of 4,184 studies. After removing duplicates, 1,174 studies remained, of which 1,071 were excluded in phase 1. A total of 103 publications were selected for full-text reading, and 81 of them were excluded for the reasons listed in Figure 1. The final sample consisted of 23 studies: 12 articles in the databases, six guidelines, four directives, and one national protocol, in addition to one master’s dissertation.

From the analysis of the 23 studies included (Chart 2), it was noted that publications were found from the year 2010 onwards, with the year 2023 having the largest number of published works, with five studies, followed by the years 2022 and 2021, both with four studies, followed by the years 2018, 2015, 2016 and 2012, with two works, and the years 2020 and 2010, which had one publication.

**Chart 2:** Characterization of studies included in the scoping review, Divinópolis, Minas Gerais, Brazil, 2024

Year/authors/title	Country	Journal or institution	LoE^^*^^
2023. Thorvaldsen *et al*. Exploring use of coercion in the Norwegian ambulance service - a qualitative study^(22)^	Norway	Scand J Trauma Resusc Emerg Med	4
2023. Häikiö *et al*. Ambulance personnel use of coercion and use of safety belts in Norway^(23)^	Norway	BMC Health Serv Res	4
2023. Moore *et al*. Mental health emergencies attended by ambulances in the United Kingdom and the implications for health service delivery: a cross-sectional study^(24)^	United Kingdom	J Health Serv Res Policy	4
2023. Santana *et al. Protocolos de Atendimentos as Urgências Psiquiátricas no Atendimento Pré-Hospitalar: Revisão Integrativa da Literatura* ^(25)^	Brazil	*Arq Ciênc Saúde Unipar*	4
2023. McDowall *et al*. Physical restraint within the prehospital Emergency Medical Care Environment: A scoping review^(26)^	South Africa	Afr J Emerg Med	4
2022. NICE. Self-harm: assessment, management and preventing recurrence^(27)^	United Kingdom	National Institute for Health and Care Excellence	5
2022. Nehme *et al*. Study of prehospital video telehealth for callers with mental health-related complaints^(28)^	Australia	Emerg Med J	5
2022. QAS (AU). Clinical Practice Guidelines: Behavioural disturbances/acute behavioral disturbance^(29)^	Australia	Queensland Ambulance Service	5
2022. QAS (AU). Clinical Practice Guidelines: Behavioural disturbances/The suicidal patient^(30)^	Australia	Queensland Ambulance Service	5
2021. Stander *et al*. Prehospital care providers’ understanding of responsibilities during a behavioural emergency^(31)^	South Africa	S Afr J Psychiatr	4
2021. Zayed *et al*. Care-pathways for patients presenting to emergency ambulance services with selfharm: national survey^(32)^	United Kingdom	Emerg Med J	4
2021. Shirzad *et al*. Development of a pre-hospital emergencies protocol for the management of suicidal patients in Iran^(33)^	Iran	BMC Emerg Med	4
2021. Ministry of Health (BR). *Atendimento pré-hospitalar em saúde mental: noções das urgências e emergências em saúde mental* ^(34)^	Brazil	Ministry of Health	4
2020. Shirzad *et al*. First line in psychiatric emergency: pre-hospital emergency protocol for mental disorders in Iran^(35)^	Iran	BMC Emerg Med	4
2018. Stam *et al*. Catch and release: evaluating the safety of non-fatal heroin overdose management in the out-of-hospital environment^(36)^	Australia	Clin Toxicol (Phila)	4
2018. NSW (AU). Mental Health Safety and Quality in NSW^(37)^	Australia	NSW Ministry of Health (AU)	5
2016. Ministry of Health (BR). *Protocolos de Intervenção para o SAMU 192 - Serviço de Atendimento Móvel de Urgência - Protocolos de Suporte Básico de Vida* ^(38)^	Brazil	Ministry of Health (BR)	5
2016. Ministry of Health (BR). *Protocolos de Intervenção para o SAMU 192 - Serviço de Atendimento Móvel de Urgência - Protocolos de Suporte Avançado de Vida* ^(39)^	Brazil	Ministry of Health (BR)	5
2015. NHS (UK). Transforming urgent care and emergency services in England (UK)^(40)^	United Kingdom	National Health System	5
2015. NSW (AU). Mental Health and Drug and Alcohol Office, Mental Health for Emergency Departments^(41)^	Australia	NSW Ministry of Health (AU)	5
2012. NSW (AU). Mental health, drug and alcohol - Emergency Department and Ambulance Monitoring^(42)^	Australia	NSW Ministry of Health (AU)	5
2012. Bonfada *et al. Concepções de profissionais de saúde do Serviço de Atendimento Móvel quanto à urgência psiquiátrica* ^(43)^	Brazil	*Rev Rene*	4
2010. Bonfada. *Serviço de Atendimento Móvel de Urgência (SAMU) e à assistência às urgências psiquiátricas* ^(44)^	Brazil	UFRN	NA^^**^^

* LoE - level of evidence;

** NA - not applicable, as this is a master’s dissertation; UFRN - Universidade Federal do Rio Grande do Norte.

In relation to the countries of publication, we found papers from various parts of the world. Australia had the highest scientific output among those selected, with seven studies, followed by Brazil with six publications, and the United Kingdom with four. Subsequently, Norway, South Africa, and Iran each had two articles.

This review also resulted in the creation of a chart (Chart 3) listing nursing care provided by nursing professionals to individuals with mental disorders in MPHC. This chart was divided into four domains: safety and protection of people with mental disorders, professionals and third parties; assessment of individuals with mental disorders; management of individuals with mental disorders; and important aspects regarding mechanical restraint. The review identified 27 nursing care practices within these domains.

**Chart 3:** Description of recommended nursing care for individuals with mental disorders in mobile prehospital care, Divinópolis, Minas Gerais, Brazil, 2024

Safety and protection of people with mental disorders, professionals and third parties
1. Pre-scene assessment of location security, escape routes, and safe locations in case of user aggression^(22,28,29,32,33,36,37)^. 2. Assess individuals’ access to weapons and equipment that could threaten their own life, the lives of professionals, and/or others^(22,28,29,32,33,36,37)^. 3. Assess the risk and need for police support, which may include anticipating police arrival to provide professional support and scene security^(28,29,32,33,36,37)^. 4. Assess risk factors for violence and anticipate it. Symptoms of imminent aggression include^(28,32,33)^: a) Motor restlessness and agitation; b) Loud and threatening tone of voice; c) Threatening behavior and gestures; d) Verbal threats; e) Staring and angry expression; f) Sudden behavior; g) Bizarre behavior due to delirium and hallucination.
**Assessment of individuals with mental disorders**
1. Assess urgent physical needs through primary assessment^(33,36,37)^. 2. Obtain patients’ targeted mental health history from family members, when reliable, including demographic characteristics (sex, age, occupation), history of psychiatric illness, history of physical and, especially, neurological illnesses, history of substance abuse, and history of violence or suicide^(28,30,33,36,37,42)^. 3. Perform differential diagnoses (psychological versus physical causes of symptoms) and physical risk factors (sudden onset of symptoms with no prior history, age under 12 and over 60, known neurological conditions such as seizures or dementia, presence of neurological symptoms such as ataxia, nystagmus, and complex medication regimen)^(28,33)^. 4. Consider cultural and spiritual aspects that may affect the therapeutic relationship during care^(28,33)^. 5. Avoid the stigmatization of people in psychological distress and the stereotypical conception that every person in crisis poses a risk to the team^(41)^.
**Management of individuals with mental disorders**
1. In behavioral management and communication with individuals experiencing mental disorders, the following are recommended^(22,28,31-33,36,37)^: a) Initiate the verbal approach by respecting individuals’ personal space of at least 2 meters away; b) Designate a mediator, taking into account individuals’ receptiveness. c) Use verbal communication in initial management, using a calm, measured, and confident tone of voice; d) Reduce external stimuli, such as noise and provocative behavior from others; e) Reduce internal triggers, such as hunger and thirst, by offering food and water to individuals whenever possible; f) Maintain empathetic and non-judgmental attitudes and behaviors; g) Accept individuals’ hallucinations and delusions appropriately; h) Be careful not to make false promises to individuals; i) Use short, simple sentences, repeating the sentences if necessary; j) Listen to individuals experiencing mental disorders; k) Reassure the person that you understand the problem; l) Encourage the person to provide information to those who can help; m) Attempt to meet the spiritual needs of the person being assisted (include general spiritual principles in the patient-therapist relationship, showing compassion and acceptance); n) Make appropriate accommodations for any learning difficulties or physical, mental, or neurodevelopmental conditions a person may have; o) Tell patients that physical aggression is unacceptable. Therefore, offer medication as a pharmacological approach, and if there is still a risk of harm to themselves and/or others, use mechanical restraint. 2. Perform pharmacological intervention only when there is no response to non-pharmacological interventions and, if necessary, use fast-acting drugs with fewer side effects^(22,28)^. 3. Use mechanical restraint only when non-pharmacological and pharmacological methods are ineffective and there is an imminent risk to life and safety^(22,28,32,37,38)^. 4. Perform mechanical restraint safely, exceptionally, using at least five professionals to perform the procedure and under the direct supervision of a nurse^(32,36,37)^. 5. Pay attention to the management of suicidal emergencies, as the expert panel suggests that this type of care requires a separate protocol. Furthermore, although poisoning and drug and alcohol withdrawal have similar symptoms to psychological emergencies, they are treated completely differently and require a separate protocol^(30,33)^. 6. Develop a shared understanding of why a person self-harmed^(26,33)^. 7. Transfer a person in psychological distress to secondary and/or tertiary care, when safe to do so, using the least restrictive means possible and ensuring compliance with all legal requirements^(28)^. 8. Monitor a person continuously after a suicide attempt or suicidal crisis^(30)^. 9. Assess risk and protective factors in cases of attempted suicide^(30,33,36,37)^. 10. Call the police in cases of death by suicide^(33)^. 11. Provide effective, timely, and appropriate communication of information about a person with a mental disorder through the healthcare system^(24,38)^.
**Important aspects regarding mechanical restraint**
1. The management of mechanical restraint includes the following recommendations^(22-26,28,32,33,36-44)^: a) Explain the reason for mechanical restraint to patients and family members; b) Do not perform the procedure as a form of punishment; c) Keep the voice calm and slow during the procedure; d) Care for victims’ head throughout the procedure; e) Check vital signs, especially the pulses of the restrained extremities; f) Use the stretcher at the lowest level during the procedure; g) Assess the level of consciousness, dehydration, skin condition, and circulation in the restrained limbs; h) Document all actions performed; i) Use the anatomical position for mechanical restraint; j) Intervene pharmacologically after mechanical restraint, without reducing the level of consciousness; k) Maintain continuous visual monitoring; l) Pay attention to the duration of restraint; m) Observe positional risk related to pressure, effort, and health status. 2. Train mobile prehospital care professionals to care for people experiencing psychological distress^(22-25,43,44)^. 3. Welcome the family^(22,44)^. 4. Pay attention to the real issues of restraint, as it is typically against patients’ will and can be considered a violation of patients’ right to refuse treatment, resulting in potential litigation^(25)^. 5. Identify and record any adverse events that occur to the restrained person^(25)^. 6. Consider the confined space presented by a moving or stationary ambulance when physically restraining the patient and the impact this may have on the manpower required during restraint^(25)^.

## DISCUSSION

This scoping review presented a small sample of studies on safe nursing care for people with mental disorders in MPHC, with a low level of evidence, i.e., publications derived from descriptive studies. It is noteworthy that the implementation of evidence-based prehospital care is becoming increasingly multifaceted, in part due to the insufficient body of scientific knowledge available for this setting.

Furthermore, this knowledge comes from research that often faces serious limitations, such as financial constraints and methodological challenges, such as the difficulty in randomizing and blinding interventions, and assessing user outcomes. Additionally, research in the prehospital setting is often challenging from an ethical perspective due to the urgency, time constraints, and the need to provide care outside the hospital setting. It is believed that, as explained in this study, the level of evidence was derived from descriptive or qualitative studies or expert opinion.

The largest scientific production in the context of this study was carried out by Australia. It is noteworthy that research that had as one of its objectives to determine who are the largest producers of scientific articles in nursing at the national level stated that the United States of America is the author of 46.03% of nursing articles, followed by Australia (9.25%), the United Kingdom (7.52%), Brazil (7.52%), and Canada (5.29%)^(45)^. This is similar to what was found in this study, since in the United States of America and Canada, MPHC is mainly staffed by paramedics, and the focus of this research was the safe nursing care for people with mental disorders at MPHC.

The pursuit of providing recommended, quality healthcare is evident through ongoing efforts across various healthcare services. Although there is limited evidence on safe nursing care for individuals with mental disorders in MPHCs, overall, research on patient safety has grown exponentially since the publication of To Err is Human: Building a Safer Health Care System, further reinforcing the urgent need for more studies on this specific topic^(46)^.

It is important to emphasize that, in order to alleviate mental distress through the prevention, care, and treatment of mental disorders and substance use disorders, and to promote and maintain the mental health of people and communities worldwide, global mental health activities need to be broad and integrate a “reformulated” mental health agenda in the 2030 Agenda for Sustainable Development^(1,10,11)^.

A psychiatric emergency is defined as an acute disturbance of behavior, thought, or mood that, if left untreated, can cause harm to both individuals and others in settings, involving a risk of death or intense distress, irreparable injuries, and requiring immediate care^(28,33,36,37,42)^. Serious conditions affecting these people include anxiety, depression, psychosis, acute behavioral disorders, intentional drug overdose, and attempted suicide^(30,33,44)^.

Suicide is a painful consequence of many psychiatric disorders and one of the most common psychiatric emergencies^(30,33,44)^. Ambulance nurses are often the first point of contact for people who self-harm, and the nature of this encounter affects both immediate outcomes and future help-seeking behavior^(30,33,37,38,43)^.

In the context of increasing demand for ambulance services, mental health emergencies are among the most difficult to respond to, and these incidents are the ones that generally require the most time on scene^(22,28,29,32,33,36-44)^. Among the many difficulties faced by emergency nurses in decision-making for people with mental disorders in emergencies are fear, insecurity, lack of specific training, stigma, limited screening capacity, stereotypical perceptions of people as dangerous, anxiety, and anger, which can compromise the safe care of people with mental disorders^(22,28,29,32,33,36-44)^. Therefore, nursing professionals at MPHC play an important role in caring for people with mental disorders.

Concerning the analysis of the safety and protection of people with mental disorders, it is necessary to promote the safety and protection of people by identifying risk situations, dealing with and mitigating potential unnecessary harm related to care, through a pre-scene assessment of the safety of the location, escape routes and safe places in case of user aggression^(28,29,32,33,36,37)^, in addition to assessing the need for police support^(28,29,32,36-44)^.

In this context, it has been demonstrated that educational courses improve knowledge, attitude, skills and self-efficacy in nursing professionals’ performance, helping them to make more assertive decisions in work settings, with the aim of ensuring safe care for users, their families and healthcare professionals^(22,32,36,37)^. On the other hand, the lack of preparation and knowledge can act as a triggering factor for violent actions, of a repressive nature and without therapeutic purpose, such as the undue request for police force and the abuse of physical and mechanical restraint^(22,28,32,36,37)^.

Regarding the assessment of people with mental disorders assisted at MPHC in emergency, the triage of people in acute psychiatric crisis can be carried out safely, using algorithms that include safety, assessment, diagnosis and management^(22-24)^.

Therefore, after the safe scene, patients’ history should be obtained and their physical condition assessed. In some circumstances, a detailed assessment may not be possible due to patients’ level of agitation and/or aggression^(28,34,35)^. Assessment, when safe, should include medical history, social history, alcohol and drug use history, physical examination when safe to do so, vital signs, sedation assessment, and consideration of cultural and spiritual aspects that may affect symptoms^(24,27)^. During assessment, nursing professionals should remain calm and monitor their own emotional response to a person with mental distress^(33,34)^.

It is important to note that it is necessary to take spiritual and cultural aspects into consideration, as this can affect the therapeutic relationship in care^(28,33)^, as well as avoiding the stigmatization of people in psychological distress and the prejudiced idea that every person in crisis represents a risk to the team^(41)^.

It is important to emphasize that it is essential to consider the possibility of an underlying clinical condition that could be potentially fatal and that is manifesting with an alteration in mental state, which could interfere with user safety^(28,31-33,36,37)^.

Regarding the management of a person in mental distress, the approach needs to focus on the implementation of verbal de-escalation, in order to mitigate the degree of agitation and/or aggression, as verbal intervention is one of the most relevant pillars in treatment^(28,31-33,36,37)^. It is a combination of strategies and techniques that include modifying settings, when possible, verbal and nonverbal communication to engage individuals and establish rapport, and working with individuals to identify appropriate and safe solutions^(31-33,36,37)^.

Verbal de-escalation is composed of ten principles, which are: respecting personal space; not being provocative; establishing verbal contact; being concise; identifying desires and feelings; listening carefully; identifying areas of agreement; establishing clear boundaries; offering choices and optimism; and assessing verbal de-escalation and considering other options if these strategies and techniques are ineffective^(28,31-33,36,37)^.

There are some peculiarities regarding the verbal approach to a person attempting self-harm. It is recommended that nursing professionals identify risk and protective factors, be clear in communication, use active listening, validate a person’s feelings and perspectives, and respect their position and circumstances^(28,31-33,36,37)^. Specialized and effective interventions that can be delivered during or immediately after the first response are vital to helping a person emerge from the crisis and prevent, among other things, further suicide attempts^(31)^.

If verbal de-escalation is not successful, pharmacological, physical and mechanical intervention should be considered in order to protect users, staff and third parties, in addition to providing treatment and care to people^(22,28,32,36,37)^.

In pharmacological management, the main focus is to calm agitated users as quickly as possible without reducing their level of consciousness. Therefore, short-acting medications are preferred over long-acting medications, and the main drug options are antipsychotics and benzodiazepines^(40-42,44)^.

Regarding mechanical restraint, it should only be used as a last resort, and it is essential that continuous visual monitoring of individuals, regular checking of vital signs, pharmacological restraint, and documentation of all procedures performed are carried out^(24,27,28,30,31)^. The recommendations for mechanical restraint and the risks involved must be observed, such as positional risk, pressure-related risk, effort-related risk, and health-related risk^(33,36-38)^.

In emergencies involving psychoactive substances, nursing professionals must be alert to associated complications/clinical complications that can be life-threatening. Although these emergencies present similar symptoms to psychological emergencies, they require completely different treatment and should therefore not be neglected^(33,36-39)^.

It is important to emphasize that the management of emergencies involving self-extermination attempts requires special consideration. Experts suggest that these emergencies require a separate protocol and initiatives specifically designed for this group to ensure safety and effectiveness^(33,36,37)^. These users should be transferred to a hospital, even if professionals consider them to be at low risk for suicide^(31)^. Furthermore, although drug and alcohol poisoning and withdrawal have similar symptoms to psychological emergencies, they require completely different treatment and require a different protocol^(26,31)^.

As for care for people who self-harm, it is recommended that the team be composed of a nursing professional, a public safety worker and a mental health specialist, in addition to using a specific risk assessment tool^(31)^.

The overall goal of treatment involving a person presenting with acute behavioral disturbance is to reduce the risk of harm to that person and to others in the setting, determine the most likely cause(s) of the acute behavioral disturbance, and, if indicated, transfer the user to definitive care using the least restrictive means possible^(28,31-33,36,37)^.

When transferring care for individuals who self-harm, the emergency department may not be the most appropriate place to care, and individuals should not be left alone after a suicide attempt or in a suicidal crisis^(30,34)^. Healthcare network services should be the priority destination for individuals in psychological crisis^(24,39)^.

Safe and efficient care requires effective, timely, and appropriate transfer of important information throughout the healthcare system^(28,31,37)^. During patients’ transport in the ambulance, nursing professionals need to: negotiate with patients about securing the stretcher’s seat belts; remove any loose objects inside the ambulance from patients’ reach; pay attention to patients’ nonverbal language; avoid bringing up new topics inside the ambulance, as these may trigger unexpected reactions; seek only to respond to patients’ requests with a helpful/supportive attitude; and never leave patients unanswered^(28,31-33,36-44)^.

People in mental health crisis should be referred to a 24/7 mental healthcare service^(37,44)^. This is particularly relevant when patients, who are already known to mental healthcare services, consent to sharing their crisis plan or suicide prevention plan with emergency services^(30)^.

For SAMU, an MPHC service in Brazil that responds to psychiatric emergencies, the practical implications of this study include providing recognized evidence in the scientific field in order to provide a systematic and coordinated approach for managers and interventionist nursing professionals, in addition to being a source that can be used in continuing training and in the development and implementation of protocols and bundles for the care of people with mental disorders.

In short, the following are protocoled as practices for safety in extra-hospital settings: user identification, clean and safe care, safe procedures, safe administration of medications and solutions, promotion of a person’s involvement with their own safety, effective communication, prevention of falls and accidents, prevention of pressure injuries, and safety in the use of technology^(22,23,25,43)^.

### Study limitations

Limitations of this scoping review include the possible existence of studies in other indexing databases. Furthermore, due to the diversity of nursing care across different study methods, it was not possible to assess the methodological quality of studies due to the methodology itself.

### Contributions to nursing

This study contributes significantly to the health field, especially nursing that works in emergency MPHC, by addressing scientific evidence on safe nursing care for people with mental disorders.

It presents information necessary for the nursing team to implement safe measures for the care of people with mental disorders at MPHC, with the participation of the SAMU continuing education centers, firefighters and military police, enabling the construction and implementation of protocols, good practice manuals, in addition to training, in order to alleviate discomfort in relation to care for psychiatric emergency situations, avoid worsening victims’ condition, minimize the distress of people and their families, prevent sequelae, or even avoid death, through adequate assistance and transportation.

## CONCLUSIONS

The nursing care recommended for people with mental disorders in the emergency MPHC, in this study, sought to reinforce the knowledge and skills of participants in areas such as risk prevention, effective communication, scene management and individuals with mental disorders, behavioral management, attention to suicidal emergencies, in addition to important aspects regarding mechanical restraint and non-pharmacological and pharmacological intervention.

Furthermore, it is important to complement recommended nursing care by considering the cultural and spiritual aspects of a person with mental disorders and their family, avoiding stigmatization and the stereotypical conception that every person in crisis poses a risk to the team, and ensuring effective and timely transfer to the appropriate healthcare service. This study is not intended to exhaust the topic; on the contrary, it seeks to open new perspectives for future research on recommended nursing care for people with mental disorders in MPHC, especially with a higher level of scientific evidence, such as retrospective cohort studies, in addition to the development of protocols and bundles to be implemented in all SAMU.

## Data Availability

The research data are available in a repository: https://doi.org/10.17605/OSF.IO/M58GT.

## References

[1] Lin X, Martinengo L, Jabir AI, Ho AHY, Car J, Atun R (2023). Scope, characteristics, behavior change techniques, and quality of conversational agents for mental health and well-being: systematic assessment of apps. J Med Internet Res.

[2] Machado DM, Veras IS, Frausino LHFC, Silva JL. (2021). Psychiatric emergency service in Federal District: interdisciplinarity, pioneering spirit and innovation. Rev Bras Enferm.

[3] Rees N, Porter A, Rapport F, Hughes S, John A. (2018). Paramedics’ perceptions of the care they provide to people who self-harm: a qualitative study using evolved grounded theory methodology. PLoS One.

[4] McCann TV, Savic M, Ferguson N, Cheetham A, Witt K, Emond K (2018). Recognition of, and attitudes towards, people with depression and psychosis with/without alcohol and other drug problems: results from a national survey of Australian paramedics. BMJ Open.

[5] Daggenvoorde TH, Gijsman HJ, Goossens PJJ. (2018). Emergency care in case of acute psychotic and/or manic symptoms: Lived experiences of patients and their families with the first interventions of a mobile crisis team: a phenomenological study. Perspect Psychiatr Care.

[6] Lubman DI, Heilbronn C, Ogeil RP, Killian JJ, Matthews S, Smith K (2020). National Ambulance Surveillance System: a novel method using coded Australian ambulance clinical records to monitor self-harm and mental health-related morbidity. PLoS One.

[7] Todorova L, Johansson A, Ivarsson B. (2021). Perceptions of ambulance nurses on their knowledge and competence when assessing psychiatric mental illness. Nurs Open.

[8] Fleury MJ, Ferland F, Farand L, Grenier G, Imboua A, Gaida F. (2025). Reasons explaining high emergency department use in patients with mental illnesses: different staff perspectives. Int J Ment Health Nurs.

[9] Fisher M, Vishwas A, Cross S, Attoe C. (2020). Simulation training for Police and Ambulance Services: improving care for people with mental health needs. BMJ Simul Technol Enhanc Learn.

[10] Moitra M, Owens S, Hailemariam M, Wilson KS, Mensa-Kwao A, Gonese G (2023). Global Mental Health: where we are and where we are going. Curr Psychiatry Rep.

[11] Collins PY. (2020). What is global mental health?. World Psychiatry.

[12] Ministério da Saúde (BR) (2011). Institui a Rede de Atenção Psicossocial para pessoas com sofrimento ou transtorno mental e com necessidades decorrentes do uso de crack, álcool e outras drogas, no âmbito do Sistema Único de Saúde.

[13] Ministério da Saúde (BR) (2017). Consolidação das normas sobre as redes do Sistema Único de Saúde.

[14] Nóbrega MPSS, Mantovani GS, Domingos AM. (2020). Resources, objectives and guidelines in a Psychosocial Care Network structure. Rev Bras Enferm.

[15] Ministério da Saúde (BR) (2013). Manual instrutivo da Rede de Atenção às Urgências e Emergências no Sistema Único de Saúde (SUS).

[16] Oliveira LC, Menezes HF, Oliveira RL, Lima DM, Fernandes SF, Silva RAR. (2020). Mobile care service for psychiatric urgencies and emergencies: perception of nursing workers. Rev Bras Enferm.

[17] Silva LLT, Dias FCS, Maforte NTP, Menezes AC. (2022). Patient safety in Primary Health Care: perception of the nursing team. Esc Anna Nery.

[18] Araujo DM, Silva ECS, Gomes HVS, Carbogim FC, Xavier GF, Coelho ACO. (2024). Leprosy and its impact on the quality of life of people with physical disabilities: a scoping review. Rev Bras Enferm.

[19] Tricco AC, Lillie E, Zarin W, O’Brien KK, Colquhoun H, Levac D (2018). PRISMA Extension for Scoping Reviews (PRISMA-ScR): checklist and Explanation. Ann Intern Med.

[20] Peters MDJ, Godfrey C, McInerney P, Munn Z, Tricco AC, Khalil H., Aromataris E, Lockwood C, Porritt K, Pilla B, Jordan Z (2024). Revisões de escopo: 2020. Manual JBI para Síntese de Evidências.

[21] The Joanna Briggs Institute (2017). Grades of recommendation: levels of evidence.

[22] Thorvaldsen NO, Husum TL, Sollid SJM. (2023). Exploring use of coercion in the Norwegian ambulance service: a qualitative study. Scand J Trauma Resusc Emerg Med.

[23] Häikiö K, Bergem AK, Holst Ø, Thorvaldsen NØ. (2023). Ambulance personnel use of coercion and use of safety belts in Norway. BMC Health Serv Res.

[24] Moore HE, Siriwardena AN, Gussy M, Spaight R. (2023). Mental health emergencies attended by ambulances in the United Kingdom and the implications for health service delivery: a cross-sectional study. J Health Serv Res Policy.

[25] Santana AA, Porcu M, Alécio R, Nacamura PAB, Ribeiro JVR, Paiano M. (2023). Protocolos de atendimentos as urgências psiquiátricas no atendimento pré-hospitalar: Revisão integrativa da literatura. Arq Ciên Saúde UNIPAR.

[26] McDowall J, Makkink AW, Jarman K. (2023). Physical restraint within the prehospital Emergency Medical Care Environment: a scoping review. Afr J Emerg Med.

[27] National Institute for Health and Care Excellence (NICE) (2022). Self-harm: assessment, management and preventing recurrence. NICE Guideline.

[28] Nehme E, Magnuson N, Mackay L, Becker G, Wilson M, Smith K. (2023). Study of prehospital video telehealth for callers with mental health-related complaints. Emerg Med J.

[29] Queensland Ambulance Service (QAS) (2022). Clinical Practice Guidelines: behavioural disturbances - acute behavioral disturbance.

[30] Queensland Ambulance Service (QAS) (2022). Clinical Practice Guidelines: behavioural disturbances - the suicidal patient.

[31] Stander C, Hodkinson P, Dippenaar E. (2021). Prehospital care providers’ understanding of responsibilities during a behavioural emergency. S Afr J Psychiatr.

[32] Zayed MG, Williams V, Glendenning AC, Bulger JK, Hewes T, Porter A (2020). Care-pathways for patients presenting to emergency ambulance services with self-harm: national survey. Emerg Med J.

[33] Shirzad F, Gholamzad S, Shafiee M, Shariat SS. (2021). Development of a pre-hospital emergencies protocol for the management of suicidal patients in Iran. BMC Emerg Med.

[34] Ministério da Saúde (BR) (2021). Atendimento pré-hospitalar em saúde mental: noções das urgências e emergências em saúde mental.

[35] Shirzad F, Hadi F, Mortazavi SS, Biglari M, Sari HN, Mohammadi Z, Atoofi MK, Shariat SV. (2020). First line in psychiatric emergency: pre-hospital emergency protocol for mental disorders in Iran. BMC Emerg Med.

[36] Stam NC, Pilgrim JL, Drummer OH, Smith K, Gerostamoulos D. (2018). Catch and release: evaluating the safety of non-fatal heroin overdose management in the out-of-hospital environment. Clin Toxicol (Phila).

[37] NSW Ministry of Health (2018). Mental Health Safety and Quality in NSW.

[38] Ministério da Saúde (BR) (2016). SAMU 192: Protocolos de suporte básico de vida.

[39] Ministério da Saúde (BR) (2016). SAMU 192: Protocolos de suporte avançado de vida.

[40] National Health System: Clinical models for ambulance services (2015). Transforming urgent and emergency care services in England.

[41] NSW Ministry of Health (2015). Mental Health and Drug and Alcohol Office, Mental Health for Emergency Departments.

[42] NSW Ministry of Health (2012). Mental Health and Drug and Alcohol Office, Mental Health for Emergency Departments.

[43] Bonfada D, Guimarães J, Brito AAC. (2012). Concepções de profissionais de saúde do serviço de atendimento móvel quanto à urgência psiquiátrica. Rev Rene.

[44] Bonfada D. (2010). Serviço de atendimento móvel de urgência (SAMU) e a assistência às urgências psiquiátricas.

[45] Silva RC, Vanz SAS. (2021). Análise da produção científica da Enfermagem e seus leitores no Mendeley. RICI.

[46] Kohn LT, Corrigan JM, Donaldson MS (2000). To Err Is Human: Building a Safer Health System. A Report of the Committee on Quality of Health Care in America.

