# Supplementary material for: Recommended nursing care for people with mental disorders in mobile prehospital care: a scoping review
Source: Rev Bras Enferm. 2025 Dec 8;78(6):e20240595. doi: 10.1590/0034-7167-2024-0595 (PMC12707532; doi:10.1590/0034-7167-2024-0595)
Supplement: Translation

# Translation: Cuidados de enfermagem recomendados às pessoas com transtornos mentais no atendimento pré-hospitalar móvel: revisão de escopo

**DOI:** 10.1590/0034-7167-2024-0595pt

**Authors:** Liliane Pena, Richardson Miranda Machado, Deborah Franscielle da Fonseca, Maria Candida Brandão Valadares Bicalho, Larissa Martins Santos, Patrícia Peres de Oliveira

**Affiliations:** I Universidade Federal de São João del-Rei. Divinópolis, Minas Gerais, Brasil

**Keywords:** Cuidados de Enfermagem, Transtornos Mentais, Assistência à, Saúde Mental, Segurança do Paciente, Serviços Médicos de Emergência.

## Abstract

**Objetivos:**

mapear as evidências científicas acerca dos cuidados de enfermagem recomendados à pessoa com transtorno mental no atendimento pré-hospitalar móvel.

**Métodos:**

revisão de escopo, conforme a metodologia JBI. Pesquisa realizada em sete bases de dados, oito catálogos de teses e dissertações, diretrizes/protocolos nacionais e *guidelines* internacionais, em qualquer idioma e sem limite temporal.

**Resultados:**

das 4.184 publicações, foram selecionadas 23, que compuseram a amostra final. Identificaram-se quatro domínios (segurança e proteção da pessoa com transtorno mental, profissionais e terceiros; avaliação da pessoa com transtorno mental; gerenciamento da pessoa em sofrimento mental; aspectos importantes quanto à contenção mecânica), subdivididos em 27 cuidados de enfermagem.

**Conclusões:**

os cuidados de enfermagem recomendados à pessoa com transtorno mental no atendimento pré-hospitalar móvel perpassaram a prevenção de riscos, comunicação eficaz, gestão da cena, manejo comportamental, atenção às emergências suicidas, além de aspectos relacionados à contenção mecânica e intervenções não farmacológicas e farmacológicas.

## INTRODUÇÃO

Os transtornos mentais afetam aproximadamente 18,0% da população global, causando uma carga substancial relacionada à saúde em todo o mundo^(1)^. Apesar de o reconhecimento de transtornos de saúde mental demandar por serviços de saúde, grandes lacunas de tratamento permanecem. Até este momento, observam-se barreiras substanciais como a falta de profissionais e recursos, percepções negativas e estigma, que dificultam comportamentos de procura por ajuda^(1,2)^.

Todos os países observaram os efeitos da pandemia de COVID-19 na saúde e no bem-estar social e emocional. Como resultado desse impacto emocional na saúde mental global, houve um aumento significativo no número de pessoas enfrentando sofrimento psíquico em todo o mundo^(1,3-5)^. Como consequência, houve um aumento nas chamadas de emergência no atendimento pré-hospitalar móvel (APHM) envolvendo pessoas com transtornos mentais, o que tem exigido que os profissionais de enfermagem socorristas realizem avaliações de crise psiquiátricas, com o conhecimento e as habilidades adequadas^(6-11)^.

Nessa perspectiva, no Brasil, a assistência à saúde mental vem passando por importantes mudanças. Atualmente, dedica-se na qualificação, efetivação e fortalecimento da Rede de Atenção Psicossocial instituída pela Portaria nº 3.088/2011^(12)^, republicada em 21 de maio de 2013 e revogada pela Portaria de Consolidação nº 3, de 28 de setembro de 2017^(13)^, que se trata da consolidação das normas sobre as Redes de Atenção à Saúde no âmbito do Sistema Único de Saúde como uma manifestação do processo político de concretização da Reforma Psiquiátrica e da Política Nacional de Saúde Mental^(14)^.

Desse modo, o cuidado em saúde mental se orienta na perspectiva das Redes de Atenção à Saúde, que o direciona por meio de diretrizes clínicas e organizativas. Os pontos de atenção de urgência e emergência são responsáveis, em seu âmbito de atuação, pelo acolhimento, classificação de risco e cuidado nas situações de urgência e emergência das pessoas com transtornos mentais e com necessidades decorrentes do uso de crack, álcool e outras drogas. Entre estes, destacam-se as Unidades Básicas de Saúde, a sala de estabilização, as portas hospitalares de atenção à urgência/pronto-socorro, a Unidade de Pronto Atendimento e o APHM^(12,13)^.

Dentro da Rede de Atenção à Urgência e Emergência^(15)^, o APHM, no Brasil, realizado pelo Serviço de Atendimento Móvel de Urgência (SAMU), tem por desígnio coordenar o fluxo assistencial e disponibilizar a assistência precoce e transporte apropriado, rápido e resolutivo às vítimas, incluindo as pessoas com transtornos mentais. Todavia, estudos apontam que, em relação ao atendimento às urgências psiquiátricas, habitualmente, o serviço reproduz a execução de atitudes que vão na contramão da Reforma Psiquiátrica e da Política Nacional de Saúde Mental, como contenções física e química efetivadas de forma equivocada e/ou em ocasiões em que não eram imprescindíveis no atendimento^(2,16)^.

Por outro lado, a assistência às pessoas em situações de urgências e emergências em saúde mental precisa ser segura, baseada em evidências científicas, usando algoritmos que incluam a avaliação, o diagnóstico e o manejo, além de ser vista como uma ocasião para propiciar acolhimento e promoção de diálogo^(2,14)^.

Apesar do avanço do conhecimento sobre segurança do paciente, a literatura ainda carece de evidências e mapeamento de cuidados recomendados de enfermagem para a pessoa em sofrimento mental no ambiente do APHM^(17)^. Ressalta-se que uma busca preliminar foi realizada no PROSPERO, na *Medical Literature Analysis and Retrieval System Online* (MEDLINE) via *National Library of Medicine and National Institutes of Health* (PubMed), *Cochrane Database of Systematic Reviews* e JBI *Evidence Synthesis*, e não foram identificadas revisões com esse enfoque concluídas ou em andamento. Portanto, esta revisão de escopo poderá contribuir para a prática de enfermagem no APHM, além do impacto potencial nas políticas de saúde e na segurança do cuidado as pessoas com transtornos mentais.

Diante do exposto, é relevante gerar evidências que possam sustentar estudos originais e sintetizar informações a respeito das urgências psíquicas no APHM, a fim de fornecer subsídios para a prática de enfermagem segura e qualificada.

Indubitavelmente, a melhoria permanente da qualidade do atendimento pré-hospitalar precisa ser desenvolvida com alicerce em evidências sólidas reconhecidas no campo científico, a fim de fornecer uma abordagem sistematizada e coordenada por parte dos gestores e profissionais de enfermagem intervencionistas.

## OBJETIVOS

Mapear as evidências científicas acerca dos cuidados de enfermagem recomendados à pessoa com transtorno mental no APHM.

## MÉTODOS

### Aspectos éticos

O uso de dados de domínio público e não envolvimento de seres humanos excluiu a necessidade de apreciação pelo Comitê de Ética em Pesquisa.

### Desenho do estudo

O presente estudo trata-se de revisão de escopo, que consiste em mapear os principais conceitos relacionados a uma área de pesquisa, bem como esclarecer as definições de trabalho e/ou limitações conceituais de um tópico com base nas evidências disponíveis, visando obter resultados abrangentes^(18-20)^.

Para garantir rigor, esta revisão de escopo foi orientada pela metodologia de revisão do JBI^(20)^, considerando-se as recomendações do *checklist Preferred Reporting Items for Systematic reviews and Meta-Analyses for Scoping Reviews* ^(19)^, além do registro de seu protocolo na plataforma *Open* Science Framework, com identificação DOI:10.17605/OSF.IO/M58GT.

Para condução do estudo, seguiram-se nove etapas: 1) definição e alinhamento do(s) objetivo(s) e identificação da questão de pesquisa; 2) desenvolvimento dos critérios de inclusão e exclusão com o objetivo; 3) descrição da abordagem planejada para busca de evidências, seleção, extração de dados e apresentação das evidências; 4) investigação das evidências; 5) seleção das evidências; 6) extração das evidências; 7) análise das evidências; 8) apresentação dos resultados; e 9) resumo das evidências em relação ao objetivo da revisão, com observação das implicações descobertas^(20)^.

### Pergunta norteadora e busca

Para a construção da pergunta de pesquisa, utilizou-se a estratégia *Participants, Concept* e *Context* (PCC)^(20)^, em que P (*Participants*) - pessoas com transtornos mentais, C (*Concept*) - cuidados de enfermagem recomendados, e C (*Context*) - SAMU. Foi estabelecida a seguinte pergunta de pesquisa: quais os cuidados de enfermagem são considerados recomendados às pessoas com transtornos mentais no contexto do APHM?

A partir da questão de pesquisa, realizou-se, inicialmente, uma pesquisa no MEDLINE via PubMed e na base de dados *Cumulative Index to Nursing and Allied Health Literature* (CINAHL) para identificar os principais descritores do *Medical Subject Headings* e Descritores em Ciências da Saúde, e palavras-chave utilizados nos estudos que abordem a temática de interesse. Ressalta-se que a estratégia de busca foi desenvolvida com a contribuição de um bibliotecário de pesquisa em ciências da saúde.

Após a seleção dos descritores e sinonímias, realizou-se a busca eletrônica dos estudos nas bases de dados eletrônicas MEDLINE via PubMed, Literatura Latino-Americana e do Caribe em Ciências da Saúde (LILACS), CINAHL, Scopus, Embase, *Web of Science* e *Cochrane Library*. A literatura cinzenta incluiu o Catálogo de Teses e Dissertações da Coordenação de Aperfeiçoamento de Pessoal de Nível Superior (CAPES), *National Library of Australia* (Trove), *Academic Archive Online* (DiVA), *The DART-Europe E-Theses Portal, National ETD Portal South African theses and dissertations, Red de Repositorios Latinoamericano, World Cat Dissertations and Theses* e Repositórios Científicos de Acesso Aberto de Portugal (RCAAP), assim como diretrizes/protocolos nacionais e *guidelines* internacionais, sem limite temporal. Como estratégia de busca dos estudos/documentos, utilizou-se a estruturação, como apresentada no Quadro 1.

**Quadro 1:** Estratégias de busca aplicadas a cada base de dados e literatura cinzenta, Divinópolis, Minas Gerais, Brasil, 2024

Bases dados	Estratégias
MEDLINE (via PubMed)	*(Mental Health OR Mental Disorders OR Psychiatry OR Intellectual Disability OR Mental Health Services OR Mental Health Assistance OR Psychiatric Nursing OR Mental Disorders Nursing OR Mental Status and Dementia Tests OR Mentally Ill Persons OR United States Substance Abuse and Mental Health Services Administration OR Persons with Mental Disabilities OR Psychic Symptoms OR Cognitive Dysfunction OR Neurocognitive Disorders OR Substance-Related Disorders OR Crisis Intervention) AND (Patient Safety OR Medication Errors OR Safety culture) AND (Ambulances OR Emergency Mobile Units OR Mobile Emergency Units OR Mobile Emergency Service OR Emergency medical assistance service OR Mobile Health Units OR Prehospital Care OR Emergency Medical Services OR Emergency Medicine OR SAMU)*
LILACS	*(Mental Health) OR (Mental Disorders) AND (Patient Safety) OR (Medication Errors) OR (Safety culture) AND (Ambulances) OR (Emergency Mobile Units) OR (Mobile Emergency Units) OR (Mobile Emergency Service) OR (Emergency medical assistance service) OR (Prehospital Care) OR (Emergency Medical Services) OR (Mobile Health Units) OR (Emergency Medicine)*
Scopus (via portal CAPES)	*(TITLE-ABS-KEY (mental AND health) OR TITLE-ABS-KEY (mental AND disorders) OR TITLE-ABS-KEY (psychiatric AND nursing) OR TITLE-ABS-KEY (mental AND disorders AND nursing) AND TITLE-ABS-KEY (patient AND safety) OR TITLE-ABS-KEY (medication AND errors) OR TITLE-ABS-KEY (safety AND culture) AND TITLE-ABS-KEY (ambulances) OR TITLE-ABS-KEY (emergency AND mobile AND units) OR TITLE-ABS-KEY (prehospital AND care) OR TITLE-ABS-KEY (mobile AND emergency AND units)) AND PUBYEAR > 1991 AND PUBYEAR < 2024*
*Cochrane Library*	*Cochrane Reviews matching (Mental Health OR Mental Disorders OR Psychiatry OR Intellectual Disability OR Mental Health Services OR Mental Health Assistance OR Psychiatric Nursing OR Mental Disorders Nursing OR Mental Status and Dementia Tests OR Mentally Ill Persons OR United States Substance Abuse and Mental Health Services Administration OR Persons with Mental Disabilities OR Psychic Symptoms OR Cognitive Dysfunction OR Neurocognitive Disorders OR Substance-Related Disorders OR Crisis Intervention) AND (Patient Safety OR Medication Errors OR Safety culture) AND (Ambulances OR Emergency Mobile Units OR Mobile Emergency Units OR Mobile Emergency Service OR Emergency medical assistance service OR Mobile Health Units OR Prehospital Care OR Emergency Medical Services OR Emergency Medicine)*
*Web of Science*	*(Mental Health OR Mental Disorders OR Psychiatry OR Intellectual Disability OR Mental Health Services OR Mental Health Assistance OR Psychiatric Nursing OR Mental Disorders Nursing OR Mental Status and Dementia Tests OR Mentally Ill Persons OR United States Substance Abuse and Mental Health Services Administration OR Persons with Mental Disabilities OR Psychic Symptoms OR Cognitive Dysfunction OR Neurocognitive Disorders OR Substance-Related Disorders OR Crisis Intervention) AND (Patient Safety OR Medication Errors OR Safety culture) AND (Ambulances OR Emergency Mobile Units OR Mobile Emergency Units OR Mobile Emergency Service OR Emergency medical assistance service OR Mobile Health Units OR Prehospital Care OR Emergency Medical Services OR Emergency Medicine OR SAMU)*
CINAHL	*(“Mental Health” OR “Mental Disorders”) AND (“Patient Safety” OR “Safety culture”) AND (“Ambulances” OR “Emergency Mobile Units” OR “Mobile Emergency Units” OR “Prehospital Care” OR “Emergency Medical Services”)*
Embase	*(‘mental disease’:ti,ab,kw AND ‘patient safety’:ti,ab,kw OR ‘safety culture’:ti,ab,kw) AND ‘emergency care’:ti,ab,kw OR ambulances:ti,ab,kw*
**Literatura cinzenta**	**Estratégias**
*The DART-Europe E-Theses Portal*	*(Mental Health OR Mental Disorders OR Psychiatry OR Psychiatric Nursing OR Substance-Related Disorders OR Crisis Intervention) AND (Patient Safety OR Medication Errors OR Safety culture) AND (Ambulances OR Emergency Mobile Units OR Mobile Emergency Units OR Mobile Emergency Service OR Emergency medical assistance service OR Mobile Health Units OR Prehospital Care)*
CAPES	*Ambulances AND Patient Safety/ Ambulances AND Mental Health/ Ambulances AND Mental Health AND Patient Safety*
RCAAP	Saúde Mental e Segurança do Paciente e Ambulâncias Saúde Mental e Segurança do paciente e Atendimento Pré-hospitalar Saúde Mental e Segurança do paciente e Assistência Pré-Hospitalar
*National ETD portal* (EThOS)	*(Mental Disorders OR Psychiatry) AND (Patient Safety OR Medication Errors OR Safety culture) AND (Ambulances OR Mobile Emergency Units OR Prehospital Care)*
*Red de Repositorios Latinoamericanos*	*(Mental Disorders OR Psychiatry) AND (Patient Safety OR Medication Errors OR Safety culture) AND (Ambulances OR Prehospital Care)*
*WorldCat dissertations and theses*	*(Mental Disorders OR Psychiatry) AND (Patient Safety OR Medication Errors OR Safety culture) AND (Ambulances OR Mobile Emergency Units OR Prehospital Care)*
TROVE	*(Mental Disorders OR Psychiatry) AND (Patient Safety OR Medication Errors OR Safety culture) AND (Ambulances OR Mobile Emergency Units OR Prehospital Care)*
DiVA	*(Mental Disorders OR Psychiatry) AND (Patient Safety OR Medication Errors OR Safety culture) AND (Ambulances OR Mobile Emergency Units OR Prehospital Care)*
QAS	*(Mental Disorders OR Psychiatry) AND (Patient Safety OR Medication Errors OR Safety culture) AND (Ambulances OR Prehospital Care)*
NICE	*(Mental Disorders OR Psychiatry) AND (Patient Safety OR Medication Errors OR Safety culture) AND (Ambulances OR Prehospital Care)*
NHS	*(Mental Disorders OR Psychiatry) AND (Patient Safety OR Medication Errors OR Safety culture) AND (Ambulances OR Prehospital Care)*
*NSW Ministry of Health* (AU)	*(Mental Disorders OR Psychiatry) AND (Patient Safety OR Medication Errors OR Safety culture) AND (Ambulances OR Prehospital Care)*
Ministério da Saúde (BR)	Saúde Mental e Segurança do paciente e Atendimento Pré-hospitalar Saúde Mental e Segurança do paciente e Assistência Pré-Hospitalar

*NHS - National Health System; NICE - National Institute for Health and Care Excellence; TROVE - National Library of Australia; DiVA - Academic Archive Online; QAS - Queensland Ambulance Service; RCAAP - Repositórios Científicos de Acesso Aberto de Portugal; CAPES - Coordenação de Aperfeiçoamento de Pessoal de Nível Superior; CINAHL - Cumulative Index to Nursing and Allied Health Literature; LILACS - Literatura Latino-Americana e do Caribe em Ciências da Saúde; MEDLINE - Medical Literature Analysis and Retrieval System Online; PubMed - National Library of Medicine and National Institutes of Health.*

As buscas foram realizadas em 02 de janeiro de 2024, sendo estabelecida nova busca, em todas as bases e fontes, em julho de 2024, por meio do acesso remoto às bases de dados, a partir do registro no portal de periódicos da CAPES via Comunidade Acadêmica Federada. Destaca-se que novos estudos foram incluídos devido à análise reversa das referências dos estudos/documentos.

### Critérios de inclusão e exclusão

A população do estudo consistiu em pesquisas relacionadas ao cuidado de enfermagem seguro à pessoa com transtorno mental, realizadas por profissionais de enfermagem no APHM e publicadas integralmente em qualquer idioma e sem limite temporal.

Houve exclusão de artigos que não contemplaram a questão de pesquisa, publicações que não apresentaram o resumo nas bases de dados utilizadas, textos completos indisponíveis *online* e artigos que comtemplem outros profissionais de saúde atuantes no APHM, assim como resumos, anais de congressos, comentários, notas prévias editoriais, resenhas, cartas, relatos de experiências, ensaios teóricos, estudos de caso único e revisões narrativas.

### Seleção, análise e tratamento dos dados

A seleção dos estudos foi conduzida por duas revisoras independentes previamente treinadas, e as divergências foram resolvidas por um terceiro revisor. O processo de seleção ocorreu em duas etapas. Na primeira, foram analisados os títulos e resumos das referências obtidas pela estratégia de busca, com a pré-seleção dos estudos potencialmente elegíveis. Na segunda etapa, realizou-se a avaliação do texto completo dos estudos pré-selecionados para confirmar sua elegibilidade, ou seja: a) confirmar sua relevância em relação à pergunta da revisão e, se for o caso, b) extrair os dados pertinentes para posterior construção deste estudo.

Ressalta-se que se utilizou como gerenciador de bibliografias o *software Mendeley Desktop*, versão 1.19.2. Os resultados obtidos foram exportados para o gerenciador de referências Rayyan^®^, desenvolvido pelo *Qatar Computing Research Institute*. O gerenciador possibilitou a retirada de documentos duplicados, a seleção e triagem dos documentos por três pesquisadores de forma independente, e as discordâncias foram resolvidas por consenso.

As informações dos documentos selecionados para análise foram extraídas de maneira independente, com o uso de planilhas do *Microsoft Excel* ^®^. Realizou-se também um teste piloto do formulário estruturado de extração de dados em cada tipo de fonte de evidência^(20)^. Um quarto revisor participou da validação das informações e da discussão para estabelecimento de consenso entre os autores, quando necessário.

Após o teste piloto, uma discussão em grupo com os autores da revisão de escopo foi realizada, a fim de chegar a um acordo sobre todos os aspectos da ferramenta, dados a serem extraídos, solução de dúvidas e conflitos.

O formulário estruturado permitiu a síntese, interpretação dos dados e análise numérica básica da extensão, natureza e distribuição dos estudos. Foram agrupados itens como os autores, o ano de publicação, o país de origem, os objetivos, o local de publicação, os cuidados de enfermagem recomendados à pessoa com transtorno mental no APHM, o nível de evidência e o *link* de acesso na íntegra. Dessa forma, em cada publicação, foram identificados e extraídos os principais aspectos relacionados à problemática, aos contextos, aos métodos, aos resultados, às discussões e às conclusões dos estudos.

Optou-se por avaliar o nível de evidência, classificado de acordo com a proposta do JBI^(21)^, sendo considerado: nível 1: estudos experimentais (revisão sistemática de ensaios clínicos randomizados controlados, ensaio clínico randomizado controlado e pseudo-ensaio clínico randomizado controlado); nível 2: estudos quase-experimentais (revisão sistemática de estudos quase-experimentais, revisão sistemática de estudos quase-experimentais e outros desenhos de menor evidência, estudo prospectivo controlado quase-experimental, pré-teste, pós-teste ou estudo de grupo controle); nível 3: estudos analíticos observacionais (revisão sistemática de estudos de coortes comparáveis, revisão sistemática de coortes comparáveis e outros desenhos de estudo de menor evidência, estudo de coorte com grupo controle, estudo caso controle e estudos observacionais sem um grupo controle); nível 4: estudos descritivos observacionais (revisão sistemática de estudos descritivos, estudo transversal, séries de casos e estudo de caso); nível 5: opinião de especialistas e pesquisas de bancada (revisão sistemática de opinião de especialistas, consenso de especialistas e pesquisa de bancada/opinião de um especialista).

O mapeamento das informações foi estabelecido com base no instrumento do JBI para caracterizar as produções^(20)^, e os estudos/documentos encontrados foram minuciosamente analisados. Os resultados foram sintetizados por meio de uma abordagem temática, sendo apresentados descritivamente por meio de tabelas e quadros.

Posteriormente, os dados foram categorizados em domínios de acordo com os cuidados de enfermagem recomendados à pessoa com transtorno mental no APHM. Com base nos dados categorizados, foi realizada uma apresentação narrativa das informações.

## RESULTADOS

A busca dos dados gerou um total de 4.184 estudos. Após a remoção das duplicatas, restaram 1.174 trabalhos, das quais 1.071 foram excluídas na fase 1. Selecionaram-se 103 publicações para leitura na íntegra, e 81 deles foram excluídos pelos motivos listados na Figura 1. A amostra final foi composta por 23 estudos, sendo 12 artigos nos bancos de dados, seis *guidelines*, quatro diretrizes e um protocolo nacional, além de uma dissertação de mestrado.

**Figura 1:** Fluxograma referente ao processo de seleção dos estudos da revisão de escopo adaptado do *Preferred Reporting Items for Systematic reviews and Meta-Analyses for Scoping Reviews* ^(20)^, Divinópolis, Minas Gerais, Brasil, 2024

A partir da análise dos 23 estudos incluídos (Quadro 2), notou-se que foram encontradas publicações a partir do ano de 2010, sendo que o ano de 2023 foi o que contou com o maior número de trabalhos publicados, possuindo cinco estudos, seguido pelos anos de 2022 e 2021, ambos com quatro estudos, em seguida dos anos de 2018, 2015, 2016 e 2012, com dois trabalhos, e dos anos de 2020 e 2010, que contaram com uma publicação.

**Quadro 2:** Caracterização dos estudos incluídos na revisão de escopo, Divinópolis, Minas Gerais, Brasil, 2024

Ano/autores/título	País	Periódico ou instituição	NE^^*^^
2023. Thorvaldsen *et al. Exploring use of coercion in the Norwegian ambulance service - a qualitative study* ^(22)^	Noruega	*Scand J Trauma Resusc Emerg Med*	4
2023. Häikiö *et al. Ambulance personnel use of coercion and use of safety belts in Norway* ^(23)^	Noruega	*BMC Health Serv Res*	4
2023. Moore *et al. Mental health emergencies attended by ambulances in the United Kingdom and the implications for health service delivery: a cross-sectional study* ^(24)^	Reino Unido	*J Health Serv Res Policy*	4
2023. Santana *et al*. Protocolos de Atendimentos as Urgências Psiquiátricas no Atendimento Pré-Hospitalar: Revisão Integrativa da Literatura^(25)^	Brasil	Arq Ciênc Saúde Unipar	4
2023. McDowall *et al. Physical restraint within the prehospital Emergency Medical Care Environment: A scoping review* ^(26)^	África do Sul	*Afr J Emerg Med*	4
2022. NICE. *Self-harm: assessment, management and preventing recurrence* ^(27)^	Reino Unido	*National Institute for Health and Care Excellence*	5
2022. Nehme *et al. Study of prehospital video telehealth for callers with mental health-related complaints* ^(28)^	Austrália	*Emerg Med J*	5
2022. QAS (AU). *Clinical Practice Guidelines: Behavioural disturbances/acute behavioral disturbance* ^(29)^	Austrália	*Queensland Ambulance Service*	5
2022. QAS (AU). *Clinical Practice Guidelines: Behavioural disturbances/The suicidal patient* ^(30)^	Austrália	*Queensland Ambulance Service*	5
2021. Stander *et al. Prehospital care providers’ understanding of responsibilities during a behavioural emergency* ^(31)^	África do Sul	*S Afr J Psychiatr*	4
2021. Zayed *et al. Care-pathways for patients presenting to emergency ambulance services with selfharm: national survey* ^(32)^	Reino Unido	Emerg Med J	4
2021. Shirzad *et al. Development of a pre-hospital emergencies protocol for the management of suicidal patients in Iran* ^(33)^	Irã	BMC Emerg Med	4
2021. Ministério da Saúde (BR). Atendimento pré-hospitalar em saúde mental: noções das urgências e emergências em saúde mental^(34)^	Brasil	Ministério da Saúde	4
2020. Shirzad *et al. First line in psychiatric emergency: pre-hospital emergency protocol for mental disorders in Iran* ^(35)^	Irã	BMC Emerg Med	4
2018. Stam *et al. Catch and release: evaluating the safety of non-fatal heroin overdose management in the out-of-hospital environment* ^(36)^	Austrália	Clin Toxicol (Phila)	4
2018. NSW (AU). *Mental Health Safety and Quality in NSW* ^(37)^	Austrália	*NSW Ministry of Health* (AU)	5
2016. Ministério da Saúde (BR). Protocolos de Intervenção para o SAMU 192 - Serviço de Atendimento Móvel de Urgência - Protocolos de Suporte Básico de Vida^(38)^	Brasil	Ministério da Saúde (BR)	5
2016. Ministério da Saúde (BR). Protocolos de Intervenção para o SAMU 192 - Serviço de Atendimento Móvel de Urgência - Protocolos de Suporte Avançado de Vida^(39)^	Brasil	Ministério da Saúde (BR)	5
2015. NHS (UK). *Transforming urgent care and emergency services in England (UK)* ^(40)^	Reino Unido	*National Health System*	5
2015. NSW (AU). *Mental Health and Drug and Alcohol Office,* *Mental Health for Emergency Departments* ^(41)^	Austrália	*NSW Ministry of Health* (AU)	5
2012. NSW (AU). *Mental health, drug and alcohol - Emergency Department and Ambulance Monitoring* ^(42)^	Austrália	*NSW Ministry of Health* (AU)	5
2012. Bonfada *et al*. Concepções de profissionais de saúde do Serviço de Atendimento Móvel quanto à urgência psiquiátrica^(43)^	Brasil	Rev Rene	4
2010. Bonfada. Serviço de Atendimento Móvel de Urgência (SAMU) e à assistência às urgências psiquiátricas^(44)^	Brasil	UFRN	NA^^**^^

* NE - nível de evidência;

** NA - não se aplica, por ser uma dissertação de mestrado; UFRN - Universidade Federal do Rio Grande do Norte.

Quanto aos países de publicação, foi possível encontrar trabalhos de vários lugares do mundo. A Austrália foi o país com maior produção científica entre os selecionados, abordando sete estudos, seguida pelo Brasil, com seis publicações, e Reino Unido, com quatro produções. Posteriormente, os países da Noruega, da África do Sul e do Irã tiveram dois artigos cada.

A presente revisão permitiu ainda a composição de um quadro (Quadro 3), listando os cuidados de enfermagem à pessoa com transtorno mental no APHM realizados por profissionais de enfermagem. Este foi dividido em quatro domínios: segurança e proteção da pessoa com transtorno mental, profissionais e terceiros; avaliação da pessoa com transtorno mental; gerenciamento da pessoa em sofrimento mental; aspectos importantes quanto à contenção mecânica. A partir da revisão, identificaram-se 27 cuidados de enfermagem dentro desses domínios.

**Quadro 3:** Descrição das cuidados de enfermagem recomendados à pessoa com transtorno mental no atendimento pré-hospitalar móvel, Divinópolis, Minas Gerais, Brasil, 2024

Segurança e proteção da pessoa com transtorno mental, profissionais e terceiros
1. Avaliar pré-cena da segurança do local, rotas de fuga e locais seguros em caso de agressividade do usuário^(22,28,29,32,33,36,37)^. 2. Avaliar o acesso da pessoa a armas e equipamentos que possam ameaçar sua própria vida, dos profissionais e/ou terceiros^(22,28,29,32,33,36,37)^. 3. Avaliar o risco e a necessidade de apoio policial, o que pode incluir a antecipação da entrada policial com a finalidade de ofertar apoio profissional e segurança de cena^(28,29,32,33,36,37)^. 4. Avaliar os fatores de risco para violência e prevê-la. Os sintomas de agressão iminente incluem^(28,32,33)^: a) Inquietação motora e agitação; b) Tom de voz alto e ameaçador; c) Comportamento e gestos ameaçadores; d) Ameaças verbais; e) Modo de olhar fixo e rosto zangado; f) Comportamentos repentinos; g) Comportamento bizarro devido delírio e alucinação.
**Avaliação da pessoa com transtorno mental**
1. Verificar as necessidades físicas urgentes por meio da avaliação primária^(33,36,37)^. 2. Obter o histórico de saúde mental direcionado do usuário por meio de familiares, quando confiável, incluindo características demográficas (sexo, idade, ocupação), histórico de doença psiquiátrica, histórico de doenças físicas e, principalmente, neurológicas, histórico de abuso de drogas, história de violência ou suicídio^(28,30,33,36,37,42)^. 3. Realizar diagnósticos diferenciais (causas psicológicas *versus* causas físicas dos sintomas) e fatores de risco físicos (início súbito dos sintomas sem história prévia, idade inferior a 12 anos e superior a 60 anos, doenças neurológicas conhecidas como convulsões ou demência, existência de sintomas neurológicos como ataxia, nistagmo e regime medicamentoso complexo)^(28,33)^. 4. Considerar aspectos culturais e espirituais que podem afetar o relacionamento terapêutico no atendimento^(28,33)^. 5. Evitar a estigmatização de pessoas em sofrimento psíquico e a concepção estereotipada de que toda pessoa em crise oferece riscos à equipe^(41)^.
**Gerenciamento da pessoa em sofrimento mental**
1. No manejo comportamental e na fala com a pessoa em sofrimento mental, recomenda-se^(22,28,31-33,36,37)^: a) Iniciar a abordagem verbal respeitando o espaço pessoal da pessoa em sofrimento mental de pelo menos 2 metros de distância; b) Definir um mediador, tendo em consideração a receptividade da pessoa com transtorno mental. c) Utilizar a comunicação verbal no manejo inicial em tom de voz calmo, medido e confiante; d) Reduzir estímulos externos, como ruído e comportamento provocativo de outras pessoas; e) Reduzir gatilhos internos, como fome e sede, oferecendo água e comida à pessoa assistida, sempre que possível; f) Ter atitudes e comportamentos empáticos e sem julgamento; g) Aceitar as alucinações e delírios do usuário de forma adequada; h) Atentar para não fazer uma falsa promessa à pessoa; i) Usar frases curtas e simples, repetindo as frases, se necessário; j) Ouvir a pessoa em sofrimento mental; k) Tranquilizar a pessoa de que você entende o problema; l) Incentivar a pessoa a fornecer informações para aqueles que podem ajudar; m) Tentar atender às necessidades espirituais da pessoa assistida (incluir princípios espirituais gerais no relacionamento paciente-terapeuta, mostrando compaixão e aceitação); n) Fazer adaptações apropriadas para qualquer dificuldade de aprendizagem ou condição física, mental ou de neurodesenvolvimento que a pessoa possa ter; o) Dizer ao usuário que a agressão física é inaceitável. Sendo assim, ofereça medicamentos como abordagem farmacológica, e caso ainda haja riscos de danos para si e/ou terceiros, utilize contenção mecânica. 2. Realizar intervenção farmacológica somente quando não houver resposta às intervenções não farmacológicas e, se for o caso, utilizar drogas de ação rápida e com menores efeitos colaterais^(22,28)^. 3. Empregar a contenção mecânica somente quando os métodos não farmacológicos e farmacológicos forem ineficazes e houver iminente risco de vida e segurança^(22,28,32,37,38)^. 4. Realizar a contenção mecânica de maneira segura, excepcionalmente, utilizando no mínimo cinco profissionais para realização do procedimento e sob supervisão direta do enfermeiro^(32,36,37)^. 5. Atentar para o gerenciamento de emergências suicidas, uma vez que o painel de especialistas sugere que este tipo de atendimento necessita de um protocolo separado. Além disso, embora o envenenamento e a privação de drogas e álcool tenham sintomas semelhantes às emergências psicológicas, eles têm um tratamento completamente diferente e precisam de outro protocolo^(30,33)^. 6. Desenvolver uma compreensão compartilhada do porquê a pessoa se automutilou^(26,33)^. 7. Transferir a pessoa em sofrimento psíquico para atendimento na atenção secundária e/ou terciária, quando for seguro fazê-lo, utilizando os meios menos restritivos possíveis e garantindo o cumprimento de todos os requisitos legais^(28)^. 8. Acompanhar ininterruptamente a pessoa após uma tentativa de suicídio ou em uma crise suicida^(30)^. 9. Avaliar os fatores de risco e proteção, em casos de tentativa de suicídio^(30,33,36,37)^. 10. Chamar a polícia em caso de óbito proveniente de suicídio^(33)^. 11. Realizar transferência eficaz, oportuna e apropriada de informações que acompanham a pessoa com transtorno mental por meio do sistema de saúde^(24,38)^.
**Aspectos importantes quanto à contenção mecânica**
1. No manejo da contenção mecânica, inclui as seguintes recomendações^(22-26,28,32,33,36-44)^: a) Explicar a pessoa assistida e aos familiares o motivo da contenção mecânica; b) Não realizar o procedimento como forma de punição; c) Manter a voz em tom calmo e lento durante o procedimento; d) Cuidar da cabeça da vítima durante todo o procedimento; e) Verificar os sinais vitais, especialmente os pulsos das extremidades sob as contenções; f) Utilizar a maca no nível mais baixo durante o procedimento; g) Avaliar quanto ao nível de consciência, desidratação, condições da pele e circulação no local dos membros contidos; h) Documentar todas as ações realizadas; i) Utilizar a posição anatômica para a contenção mecânica; j) Intervir farmacologicamente após a contenção mecânica, sem reduzir o nível de consciência; k) Manter monitoramento visual contínuo; l) Atentar quanto ao limite de duração da contenção; m) Observar quanto ao risco posicional relacionado à pressão, esforço e estado de saúde. 2. Capacitar os profissionais do atendimento pré-hospitalar móvel para o cuidado da pessoa em sofrimento psíquico^(22-25,43,44)^. 3. Acolher a família^(22,44)^. 4. Atentar para as questões reais da contenção, pois é tipicamente contra a vontade dos usuários e pode ser considerada uma violação do direito das pessoas de recusar o tratamento, resultando em potencial litígio^(25)^. 5. Identificar e registrar caso ocorram eventos adversos da pessoa contida^(25)^. 6. Considerar o espaço confinado apresentado por uma ambulância em movimento ou estacionária ao conter fisicamente o usuário e o efeito que isso pode ter sobre a mão de obra necessária durante a contenção^(25)^.

## DISCUSSÃO

A presente revisão de escopo apresentou uma amostra reduzida de estudos sobre o cuidado de enfermagem seguro à pessoa com transtorno mental no APHM, com baixo nível de evidência, ou seja, publicações derivadas de estudo descritivos. Ressalta-se que a implementação dos cuidados pré-hospitalares baseados em evidências está se tornando cada vez mais multifacetada, em parte devido ao insuficiente corpo de conhecimento científico disponível para esse ambiente.

Além disso, esse conhecimento advém de pesquisas que, frequentemente, enfrentam sérias limitações, como restrições financeiras e desafios metodológicos, como a dificuldade em randomizar e cegar intervenções, avaliando os resultados dos usuários. Adicionalmente, a pesquisa no contexto pré-hospitalar é muitas vezes difícil do ponto de vista ético, devido à urgência, à limitação de tempo e à necessidade de realizar os cuidados fora do ambiente hospitalar. Acredita-se que, conforme explicado neste estudo, o nível de evidência foi derivado de estudos descritivos ou qualitativos ou de opinião de especialistas.

A maior produção científica no contexto deste estudo foi realizada pela Austrália. Ressalta-se que pesquisa que teve como um de seus objetivos apurar quem são os maiores produtores de artigos científicos da enfermagem em nível de país afirmou que os Estados Unidos da América são autores de 46,03% dos artigos de enfermagem, seguidos por Austrália (9,25%), Reino Unido (7,52%), Brasil (7,52%) e Canadá (5,29%)^(45)^, similar ao do encontrado neste estudo, uma vez que nos Estados Unidos da América e no Canadá, no APHM, trabalham, principalmente, paramédicos, e o foco desta pesquisa foi o cuidado de enfermagem seguro à pessoa com transtorno mental no APHM.

A busca pela oferta de cuidados em saúde recomendados e de qualidade é observada por meio dos esforços contínuos em diferentes serviços de saúde. Apesar de existirem poucas evidências sobre o cuidado de enfermagem seguro à pessoa com transtorno mental no APHM, no contexto geral, as investigações sobre a segurança do paciente têm crescido exponencialmente desde a publicação do *To Err is Human: Building a Safer Health Care System*, o que reforça ainda mais sobre a necessidade urgente de mais estudos que envolvam esta temática específica^(46)^.

Importante salientar que, a fim de aliviar o sofrimento mental por meio da prevenção, cuidado, tratamento de transtornos mentais e de uso de substâncias psicoativas, e promover e manter a saúde mental de pessoas e comunidades em todo o mundo, atividades globais de saúde mental precisam ser amplas e integrar uma agenda de saúde mental “reformulada” na Agenda 2030 para o Desenvolvimento Sustentável^(1,10,11)^.

A emergência psiquiátrica é definida como uma perturbação aguda do comportamento, pensamento ou humor que, se não tratada, pode causar danos, tanto ao sujeito quanto a outras pessoas no ambiente, que implicam risco de morte ou sofrimento intenso, lesões irreparáveis e necessita de atendimento imediato^(28,33,36,37,42)^. Os quadros graves que envolvem essas pessoas incluem ansiedade, depressão, psicose, distúrbios comportamentais agudos, overdose intencional de drogas e tentativa de suicídio^(30,33,44)^.

O suicídio é uma consequência dolorosa de muitos transtornos psiquiátricos e uma das emergências psiquiátricas mais frequentes^(30,33,44)^. Os profissionais de enfermagem que atuam na ambulância são frequentemente o primeiro ponto de contato para pessoas que se automutilam, e a natureza desse encontro afeta os resultados imediatos e o comportamento futuro da procura por ajuda^(30,33,37,38,43)^.

No contexto da crescente procura de serviços de ambulância, os casos de emergência de saúde mental estão entre os mais difíceis de serem atendidos, e essas ocorrências são as que geralmente requerem mais tempo na cena^(22,28,29,32,33,36-44)^. Entre muitas dificuldades dos profissionais de enfermagem socorristas na tomada de decisão para as pessoas em urgência envolvendo transtorno mental, podem-se citar o medo, a insegurança, falta de treinamento específico, estigma, limitação na capacidade de triagem, periculosidade das pessoas como concepção estereotipada, ansiedade e raiva, o que pode comprometer o atendimento seguro à pessoa com transtorno mental^(22,28,29,32,33,36-44)^. Destarte, o profissional de enfermagem do APHM tem um papel importante na assistência da pessoa com transtorno mental.

Em relação à análise quanto à segurança e proteção da pessoa com transtorno mental, é necessário promover a segurança e proteção da pessoa ao identificar situações de risco, lidar e amenizar potenciais danos desnecessários ligados ao atendimento, por meio da avaliação pré-cena da segurança do local, rotas de fuga e locais seguros em caso de agressividade do usuário^(28,29,32,33,36,37)^, além da avaliação da necessidade de apoio policial^(28,29,32,36-44)^.

Nesse contexto, foi demonstrado que os cursos educacionais aprimoram o conhecimento, a atitude, as habilidades e a autoeficácia no desempenho dos profissionais de enfermagem, auxiliando-os na tomada de decisão mais assertiva no ambiente de trabalho, com o objetivo de garantir um atendimento seguro para os usuários, seus familiares e os profissionais de saúde^(22,32,36,37)^. Por outro lado, a falta de preparo e conhecimento pode atuar como um fator desencadeador de ações violentas, de natureza repressiva e sem finalidade terapêutica, como a solicitação indevida de força policial e o abuso da contenção física e mecânica^(22,28,32,36,37)^.

No que concerne à avaliação da pessoa com transtorno mental assistida na APHM em urgência, a triagem das pessoas em crise aguda psiquiátrica pode ser realizada com segurança, usando os algoritmos que incluam segurança, avaliação, diagnóstico e manejo^(22-24)^.

Assim, após a cena segura, deve-se obter a história da pessoa e avaliar a condição física do mesmo, em algumas circunstâncias, uma avaliação detalhada pode não ser possível devido ao grau de agitação e/ou agressividade do usuário^(28,34,35)^. A avaliação, quando segura, deve incluir história clínica, história social, história do consumo de álcool e drogas, exame físico, quando for seguro fazê-lo, sinais vitais, avaliação de sedação, e considerar os aspectos culturais e espirituais que podem afetar os sintomas^(24,27)^. Durante a avaliação, o profissional de enfermagem deve se manter calmo e monitorar a sua própria resposta emocional em relação à pessoa em sofrimento mental^(33,34)^.

Ressalta-se que é necessário levar em consideração aspectos espirituais e culturais, uma vez que pode afetar o relacionamento terapêutico na assistência^(28,33)^, bem como evitar a estigmatização de pessoas em sofrimento psíquico e a ideia preconceituosa de que toda pessoa em crise representa um risco para a equipe^(41)^.

Importante salientar que é imprescindível considerar a possibilidade de uma condição clínica subjacente que possa ser potencialmente fatal e que esteja manifestando com alteração do estado mental, podendo interferir na segurança do usuário^(28,31-33,36,37)^.

No que diz respeito ao gerenciamento da pessoa em sofrimento mental, a abordagem precisa se concentrar na implementação do descalonamento verbal, a fim de mitigar o grau de agitação e/ou agressividade da mesma, pois a intervenção verbal é um dos pilares de maior relevância no tratamento^(28,31-33,36,37)^. É uma combinação de estratégias e técnicas que incluem a modificação do ambiente quando possível, comunicação verbal e não verbal para envolver a pessoa e estabelecer um relacionamento e trabalhar com a pessoa, a fim de identificar soluções apropriadas e seguras^(31-33,36,37)^.

O descalonamento verbal é composto por dez princípios, sendo estes: respeitar o espaço pessoal; não ser provocativo; estabelecer contato verbal; ser conciso; identificar desejos e sentimentos; ouvir atentamente; identificar áreas sobre as quais concordar; estabelecer limites claros; oferecer escolhas e otimismo; e avaliar a desescalada verbal e considerar outras opções em caso de ineficácia dessas estratégias e técnicas^(28,31-33,36,37)^.

Há algumas peculiaridades em relação à abordagem verbal da pessoa em tentativa de autoextermínio. É recomendado que o profissional de enfermagem identifique os fatores de risco e proteção, seja claro na comunicação, use a escuta ativa, valide os sentimentos e perspectivas da pessoa, e respeite a posição e circunstâncias da mesma^(28,31-33,36,37)^. Intervenções especializadas e eficazes que possam ser realizadas durante ou imediatamente após a primeira resposta são vitais para ajudar uma pessoa a sair da crise e prevenir, entre outras coisas, novas tentativas de suicídio^(31)^.

Se o descalonamento verbal não for bem-sucedido, deve-se considerar a intervenção farmacológica, física e mecânica, com o intuito de proteger o usuário, a equipe e terceiros, além de fornecer tratamento e cuidados à pessoa^(22,28,32,36,37)^.

No manejo farmacológico, o foco principal é tranquilizar o usuário agitado o mais rápido possível sem reduzir o nível de consciência. Sendo assim, os medicamentos de ação curta são preferidos aos medicamentos de ação prolongada, e as principais opções medicamentosas são os antipsicóticos e os benzodiazepínicos^(40-42,44)^.

Em relação à contenção mecânica, deve ser utilizada somente como último recurso, e é essencial que seja realizado o monitoramento visual contínuo da pessoa, verificação regular dos sinais vitais, contenção farmacológica e documentação de todos os procedimentos realizados^(24,27,28,30,31)^. Devem-se observar as recomendações na contenção mecânica e os riscos que envolvem a mesma, como o risco posicional, o relacionado à pressão, o relacionado ao esforço e o relacionado ao estado de saúde^(33,36-38)^.

Nos casos de urgências envolvendo substâncias psicoativas, o profissional de enfermagem precisa estar atento quanto às complicações/agravos clínicos associados que podem implicar risco de morte. Apesar de elas apresentarem sintomas semelhantes aos das emergências psicológicas, elas têm um tratamento completamente diferente e, portanto, não devem ser negligenciadas^(33,36-39)^.

Ressalta-se que o manejo de emergências que envolvem as tentativas de autoextermínio requer consideração especial. Os especialistas sugerem que essas emergências precisam de um protocolo separado e iniciativas relevantes para este grupo, a fim de assegurar a segurança e a efetividade^(33,36,37)^. Esses usuários devem ser transferidos para um hospital, mesmo que o profissional considere que os mesmos apresentam baixo risco para o suicídio^(31)^. Além disso, embora o envenenamento e a privação de drogas e álcool tenham sintomas semelhantes às emergências psicológicas, eles têm um tratamento completamente diferente e precisam de outro protocolo^(26,31)^.

No tocante ao atendimento às pessoas que se automutilam, indica-se que a equipe seja composta por profissional de enfermagem, trabalhador da segurança pública e especialista em saúde mental, além da utilização de uma ferramenta específica de avaliação de risco^(31)^.

O objetivo global do tratamento envolvendo uma pessoa que apresenta distúrbio comportamental agudo é reduzir o risco de danos a essa pessoa e a outros indivíduos no local, determinar a(s) causa(s) mais provável(is) do distúrbio comportamental agudo e, se indicado, transferir o usuário para os cuidados definitivos, utilizando os meios menos restritivos possíveis^(28,31-33,36,37)^.

Ao fazer a transferência de cuidados de pessoas que se autoagridem, o departamento de emergência pode não ser o local de atendimento mais adequado, e esses não devem ficar sozinhos após uma tentativa de suicídio ou em uma crise suicida^(30,34)^. Os serviços da rede de assistência em saúde devem ser o destino prioritário para as pessoas em crise psíquica^(24,39)^.

O atendimento seguro e eficiente à pessoa requer uma transferência eficaz, oportuna e apropriada de informações importantes que acompanham o mesmo por meio do sistema de saúde^(28,31,37)^. Durante o transporte do usuário na ambulância, o profissional de enfermagem necessita: negociar com o mesmo a fixação dos cintos de segurança da maca; retirar do alcance da pessoa quaisquer objetos soltos que fiquem dentro da ambulância; manter atenção à linguagem não verbal da pessoa; evitar novos assuntos dentro da ambulância, pois esses podem incitar reações inesperadas; buscar apenas responder solicitações da pessoa com postura de ajuda/apoio; e jamais o deixar sem resposta^(28,31-33,36-44)^.

As pessoas em crise de saúde mental devem ser encaminhadas para um serviço de saúde mental que funcione 24 horas por dia, sete dias por semana^(37,44)^. É particularmente pertinente quando o usuário, que já é conhecido pelos serviços de saúde mental, consente compartilhar o plano de crise ou plano de prevenção de suicídio com os serviços de atendimento de urgência^(30)^.

Para o SAMU, serviço de APHM, no Brasil, que atende emergências psiquiátricas, as implicações práticas deste estudo incluem fornecer evidências reconhecidas no campo científico, a fim de fornecer uma abordagem sistematizada e coordenada para os gestores e os profissionais de enfermagem intervencionistas, além de serem uma fonte que pode ser utilizada na capacitação contínua e no desenvolvimento e implementação de protocolos e *bundles* para o atendimento à pessoa com transtorno mental.

Em suma, são protocolados como práticas para a segurança no ambiente extrahospitalar a identificação do usuário, cuidado limpo e seguro, procedimento seguro, administração segura de medicamentos e soluções, promoção do envolvimento da pessoa com sua própria segurança, comunicação efetiva, prevenção de quedas e acidente, prevenção da lesão por pressão, e segurança na utilização da tecnologia^(22,23,25,43)^.

### Limitações do estudo

Reconhece-se como limitações no processo desta revisão de escopo a possível existência de estudos em outras bases de indexação. Além disso, devido à diversidade de cuidados de enfermagem em diferentes métodos de estudo, não foi possível avaliar a qualidade metodológica das pesquisas devido à própria metodologia.

### Contribuições para a área da enfermagem

O presente estudo contribui de forma significativa para a área da saúde, sobretudo a enfermagem que atua APHM de urgência, por abordar evidências científicas sobre o cuidado seguro de enfermagem à pessoa com transtorno mental.

Apresenta informações necessárias à equipe de enfermagem para a implantação de medidas seguras para o cuidado da pessoa com transtorno mental no APHM, com a participação dos núcleos de educação permanente dos SAMU, bombeiros e polícia militar, possibilitando a construção e implementação de protocolos, manuais de boas práticas, além de treinamentos, a fim de amenizar o desconforto em relação ao atendimento às situações de urgência psiquiátricas, evitar o agravamento da condição da vítima, minimizar o sofrimento da pessoa e sua família, prevenir sequelas, ou mesmo evitar o óbito, por meio de uma assistência e transporte adequados.

## CONCLUSÕES

Os cuidados de enfermagem recomendados para a pessoa com transtorno mental no APHM de urgência, neste estudo, procuraram reforçar os conhecimentos e competências dos participantes em áreas como a prevenção de riscos, comunicação eficaz, gestão da cena e da pessoa com transtorno mental, manejo comportamental, atenção às emergências suicidas, além de aspectos importantes quanto à contenção mecânica e intervenção não farmacológica e farmacológica.

Além disso, é relevante complementar os cuidados recomendados de enfermagem, por meio da consideração dos aspectos culturais e espirituais da pessoa em sofrimento mental e sua família, evitando a estigmatização e a concepção estereotipada de que toda pessoa em crise oferece riscos à equipe, e realizando transferência eficaz e oportuna para o serviço de saúde apropriado. Este estudo não tem a intenção de esgotar o tema; pelo contrário, busca abrir novas perspectivas para futuras pesquisas sobre os cuidados de enfermagem recomendados à pessoa com transtorno mental no APHM, principalmente com maior nível de evidência científica, como estudos de coorte retrospectivo, além da elaboração de protocolos e *bundles* a serem implantados em todos os SAMU.

## Data Availability

Os dados de pesquisa estão disponíveis em repositório: https://doi.org/10.17605/OSF.IO/M58GT.
